# The Potential of Cognitive‐Inspired Neural Network Modeling Framework for Computer Vision

**DOI:** 10.1002/advs.202507730

**Published:** 2025-08-19

**Authors:** Guorun Li, Lei Liu, Xiaoyu Li, Yuefeng Du, Zhenghe Song, Xiuheng Wu

**Affiliations:** ^1^ College of Engineering China Agricultural University Beijing 100083 China

**Keywords:** cognitive modeling, fast fourier transform, human memory, vision deep neural networks, visual cognitive

## Abstract

Vision deep neural networks (VDNNs) only simulate the attention‐based significance selection function in human visual perception, rather than the full spectrum of visual cognition, reflecting the divide between cognitive science (CS) and artificial intelligence (AI). To address this problem, this work proposes a cognitive modeling framework (CMF) comprising three stages: functional abstraction, operator structuring, and program agent. Then, this work defines the prior information of basic image features as the long‐term memory content in VDNNs. Meanwhile, this work introduces a memory modeling method for VDNNs based on the fast Fourier transform (FFT) and statistical methods—the unbiased mapping algorithm (UMA). Finally, this work develops visual cognitive neural units (VCNUs) and a baseline model (VCogM) based on CMF and UMA, and conduct performance testing experiments on different datasets such as natural scene recognition and agricultural image classification. The results show that VCogM and VCNU achieved state‐of‐the‐art (SOTA) performance across various recognition tasks. The model's learning process is independent of data distribution and scale, fully demonstrating the rationality of cognitive‐inspired modeling principles. The research findings provide new insights into the deep integration of CS and AI.

## Graphical abstract

In article number 202507730, Guorun Li, Lei Liu, Yuefeng Du, and co‐workers present a cognitive modeling framework (CMF) to bridge the ‘representation gap’ and ‘conceptual gap’ between cognitive theory and vision deep neural networks (VDNNs). The research findings provide new insights and solid theoretical support for VDNN modeling inspired by cognitive theory.

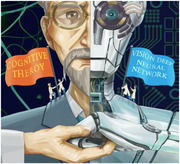

## 1 Introduction

**Figure 1:**
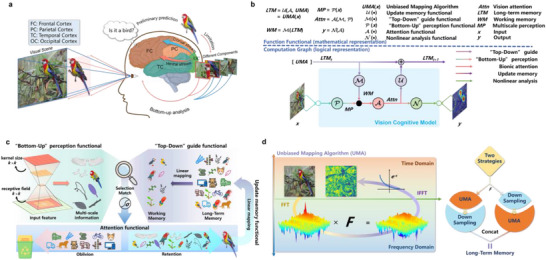
Illustration of the cognitive modeling framework (CMF). a) Illustration of the cognitive process for visual attention in the human brain. This process begins with visual stimuli formed on the retina from the external visual field. These stimuli are transmitted via the optic nerve to the primary visual cortex and processed through the ventral and dorsal pathways, leading to higher‐level visual areas where complex stimuli are formed. During this process, neurons’ receptive fields gradually enlarge and bias salient stimuli, constituting a bottom‐up automatic selection process. Higher‐level visual stimuli serve as cues to recall relevant prior knowledge from LTM, forming WM that suppresses irrelevant neural activities and enhances specific location and feature activities. This constitutes a top‐down active search process based on prior knowledge. These processes couple to form visual attention, allowing the brain to accurately distinguish objects like birds from complex backgrounds without focusing on irrelevant details. b) The CMF consists of three steps: functional abstraction, operator structuring, and program agent. The three steps of CMF introduce three concepts: function functional, computation graph, and agency program. These concepts allow cognitive functions and the causal logic between them to be embedded into the computational process of deep models, serving as explicit constraints on the operator computation principles and learning objectives. c) An example of instantiating the agency program. We instantiate the function functionals in the computation graph using convolutional operators, linear mappings, and activation functions. d) The proposed Unbiased Mapping Algorithm (UMA) provides input‐independent prior representations using Fast Fourier Transform (FFT) and statistical methods to model LTM. UMA performs low‐pass filtering in the frequency domain, then applies an *e^‐x^* function mapping to retain contour features while blurring texture features. Statistical methods define the filter. Two strategies are formulated to introduce more prior representations into LTM.

In vision deep neural networks (VDNNs), simulating the information selection mechanisms of human attention helps the model to extract valuable pixels from massive pixel matrices, thereby facilitating accurate and efficient image understanding.^[^ ^1^, ^2^ ^]^ This concept has driven the rapid development of VDNNs over the past decade,^[^ ^3^ ^]^ showcasing outstanding performance in natural object recognition,^[^ ^4^ ^]^ agricultural target classification,^[^ ^5^ ^]^ and medical auxiliary diagnosis tasks.^[^ ^6^ ^]^ However, VDNN modeling methods that focus on extracting per‐pixel weights and biases through architectural design merely simulate the selective aspect of human attention from an engineering perspective and fall far short of mapping the full spectrum of human visual attention.^[^ ^7^ ^]^ For instance, in Transformers^[^ ^8^ ^]^ and convolutional neural networks (CNNs),^[^ ^9^ ^]^ the element correlation matrix formed by matrix operations on triplets and the sliding window of the convolution operator achieves weighted averaging, respectively, with its design principles reflecting data‐driven non‐linear fitting.^[^ ^10^ ^]^ Human visual attention is a complex cognitive system involving multiple cognitive mechanisms,^[^ ^11^ ^]^ including multi‐scale perception, long‐term memory (LTM), and working memory (WM).^[^ ^12^, ^13^, ^14^ ^]^ At the same time, the biased competition theory (BCT)^[^ ^15^ ^]^ posits that attention formed by both bottom‐up (perception‐driven) and top‐down (goal‐oriented) attention mechanisms (**Figure** **1**a). Two attention mechanisms interact during the cognitive process and form the final attention bias.^[^ ^16^ ^]^ Therefore, the attention mechanisms in existing VDNNs only simulate the single process of perception‐driven attention, ignoring other cognitive functions and the interactions between these functions.

Researchers in cognitive psychology reported that LTM and WM promote the formation of top‐down attention.^[^ ^13^, ^17^, ^18^, ^19^ ^]^ For instance, embedded‐processes theory (EPT)^[^ ^13^, ^18^, ^20^ ^]^ suggests that in visual processing tasks, the brain uses various features extracted from visual pathways as visual cues to recall or retrieve information related to the current scene from LTM, generate WM, and regulate cognitive resources in the form of visual templates, directing cognitive resources to areas and features that match the templates.^[^ ^21^, ^22^ ^]^ Moreover, sustained focus on a target or scene enhances the encoding and storage quality of that target's LTM in the brain, thereby increasing the likelihood of subsequent retrieval and recall.^[^ ^13^, ^18^, ^23^ ^]^ With the emergence of the unified theory of cognition (UTC),^[^ ^24^ ^]^ cognitive scientists have integrated EPT and BCT to construct a closed‐loop visual attention system based on perception–memory coupling.^[^ ^25^, ^26^ ^]^ The above cognitive theories all demonstrate the importance of memory in human cognitive processes. However, modeling memory mechanisms remains difficult due to the lack of a clear concept of memory in VDNNs.

In natural language processing, continual learning, and time series analysis fields, exploring ways to enhance the model's learning and reasoning capabilities through memory mechanisms has been a focal point for researchers,^[^ ^27^, ^28^ ^]^ leading to notable achievements like recurrent neural network (RNN)^[^ ^29^ ^]^ and long short‐term memory (LSTM).^[^ ^30^ ^]^ The core of these models involves defining suitable historical information as memory and then constructing specific encoding and retrieval methods to constrain and recall historical information.^[^ ^31^, ^32^, ^33^ ^]^ However, the primary tasks in computer vision (CV) focus on analyzing a single image,^[^ ^34^ ^]^ which lack explicit temporal characteristics. Temporal historical information serves as the fundamental basis for defining memory in CV, making the question of what constitutes VDNNs’ memory an open issue. Recently, some studies have attempted to describe memory as pixels in serialized images, forcing pixels to possess temporal features (Figure 3b).^[^ ^35^, ^36^, ^37^ ^]^ Yet, this approach does not align with the nature of human visual memory, which encompasses more complex and abstract representations beyond simple pixel sequences.^[^ ^38^ ^]^ The limitations of VDNNs and their underlying operators in simulating visual attention and memory lead to an overreliance on the scale and quality of training data.

Simulating complex cognitive systems in VDNN is a huge challenge. This is mainly because cognitive theories are typically represented as highly abstract concept graphs, consisting of boxes that denote cognitive functions and arrows that illustrate their interactive logic.^[^ ^13^, ^17^, ^18^, ^20^ ^]^ These graphs are used to faithfully match the outcomes of human experiments and to explain the principles of cognition.^[^ ^39^, ^40^ ^]^ However, these theories lack explicit mathematical formulations or computational processes,^[^ ^20^ ^]^ allowing only partial qualitative predictive analysis.^[^ ^40^ ^]^ In contrast, VDNNs emphasize the automatic learning of underlying logic and patterns from large‐scale training data through numerical computations, thereby replicating extending human visual cognitive capabilities on computational devices.^[^ ^41^, ^42^ ^]^ The divergence in objectives between cognitive theories and VDNNs reflects the significant gap between cognitive science (CS) and artificial intelligence (AI). Currently, there is a lack of a theoretical framework for transitioning from cognitive theory to VDNNs modeling methods. Therefore, using cognitive theory concept maps to guide the design of VDNNs still presents many open and challenging issues, including functional abstraction, numerical modeling, and rule constraints.

**Figure 2:**
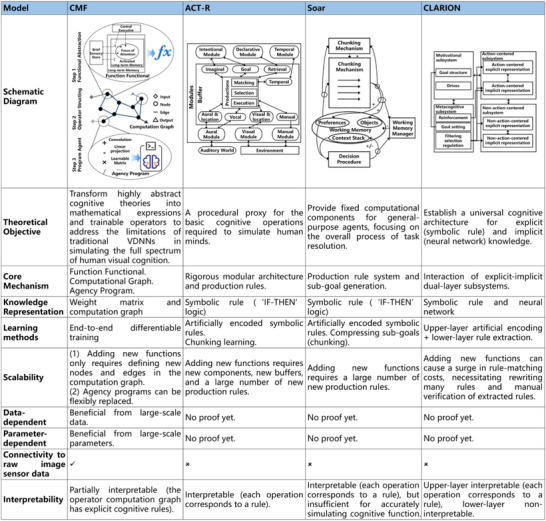
The explicit contrast of CMF with classical cognitive architectures (models).

To bridge cognitive theory and modeling VDNNs, we proposed a cognitive modeling framework (CMF). CMF is a modeling theory that integrates the functions and causal logics of various components in cognitive theory into the basic operators of VDNNs (**Figure** **2**). It aims to provide a reasonable and feasible framework for the cognitive‐inspired modeling of these operators, ensuring that VDNNs benefit from large‐scale training data and possess cognitive rationality at the conceptual level. To address the memory modeling challenge in VDNNs, we drew inspiration from the concept of visual templates^[^ ^13^, ^20^, ^43^ ^]^ and defined VDNNs’ memory as prior information composed of fundamental features in images, primarily including contours and textures, focusing on contour information. We then developed an LTM modeling method called the Unbiased Mapping Algorithm (UMA), which extracts prior information from images unsupervised using the Fast Fourier Transform (FFT)^[^ ^44^ ^]^ and statistical methods from large image datasets (Figure 1d). Compared to the previously mentioned pixels, prior information represents a more cognitively plausible form of memory, simulating the fuzziness,^[^ ^13^, ^18^ ^]^ retrievability,^[^ ^17^ ^]^ and imagery‐oriented nature of LTM,^[^ ^13^, ^20^, ^43^ ^]^ and providing a more robust and meaningful representation of memory within VDNNs.

Based on CMF and UMA, we developed the visual cognitive model (VCogM) and visual cognitive neural unit (VCNU) and applied them to specific visual processing tasks such as natural scene recognition and agricultural object recognition. Then, we integrated the Prior‐Based Memory (PBM) framework proposed in VCNU with representative VDNN operators to demonstrate the scalability and flexibility of our proposed CMF and memory in diverse image processing tasks. More importantly, our models exhibited learning and functional characteristics consistent with the human visual system. This consistency provided quantifiable guidance for further exploration of cognitive mechanisms in the brain and promises broad implications in cognitive science.

## 2 Results

### 2.1 The Cognitive Modeling Framework

CMF is a theoretical framework for modeling cognitive mechanisms in the VDNN operator (Figure 2), such as memory and attention, which specifically includes the following three stages:

**Step 1: Functional Abstraction**. Inspired by the UTC, a closed‐loop cognitive system composed of one or more cognitive theories is abstracted into several “function functionals (FFs)” based on individual cognitive components (sub‐functions), such as attention filtering, memory retrieval, memory updating, and forgetting. The FF is a formalized representation of a cognitive component through generic functionals (such as mathematical mappings *f*(*X*)). Each FF describes a cognitive sub‐function's input‐output mapping, defining different cognitive sub‐functions for operators.
**Step 2: Operator Structuring**. Based on cognitive logic, such as how attention is guided by prior knowledge and how LTM is activated, the sequence relationship between the outputs and inputs of each FF is defined. Based on this sequence relationship, the network topology structure and information flow pipeline within the operator is designed to follow cognitive logic at the level of computational principles, named computation graph. Step 2 constructs a differentiable computation graph by limiting the data flow direction and functional execution order within operators, thereby mapping the operational and collaborative logic of sub‐functions in cognitive theory. Nodes in the computation graph represent FFs, while edges represent information flow pipelines.
**Step 3: Program agent**. We develop agency programs for each FF using quantifiable operational rules or machine learning methods. This step converts the FFs from qualitative analysis into distinct numerical and differentiable calculation methods, enabling end‐to‐end optimization of cognitive functions through backpropagation.

CMF serves as a bridge by translating highly abstract cognitive theories, such as BCT and EPT, into mathematical representations and trainable operators. In CMF, Step 1 and Step 2 achieve engineering simplification of cognitive theories by retaining only core mechanisms, such as retaining only the mechanism in EPT where LTM selective activation generates WM (Figure 2); Step 3 provides independent algorithmic representations of specific cognitive functions.

CMF and traditional cognitive architecture from CS are based on cognitive theory, mapping mechanisms such as attention and memory into executable agent programs. However, CMF emphasizes the theoretical transition from human visual cognition to VDNN modeling methods. The ACT‐R,^[^ ^45^ ^]^ Soar,^[^ ^46^ ^]^ and CLARION^[^ ^47^ ^]^ cognitive architectures reflect theoretical directions for understanding human cognition from different perspectives (Figure 2). ACT‐R emphasizes explaining the biological mechanisms underlying cognition; Soar emphasizes simulating human capabilities in processing various tasks; CLARION emphasizes simulating the processing mechanisms of implicit and explicit subsystems in human cognition. These cognitive architectures hold symbolic interpretability and module interaction advantages but struggle to support large‐scale, autonomous learning and flexible scalability. Conversely, operators developed based on CMF can be implemented through existing deep learning architectures, particularly through gradient‐based backpropagation algorithms for parameter optimization, and benefit from large‐scale training data.

Within the UTC, the BCT and EPT form a closed‐loop visual cognitive system. In Step 1, five FFs are used to model the visual cognitive system. Among them, P is used to perform multi‐scale perception of the input to form bottom‐up attention; M is used to generate WM activated by LTM to form top‐down attention; A is the coupling of two attentional mechanisms; U is to encode the content of attention into LTM; N is a non‐linear analysis of attention (Figure 1b). In Step 2, A, P, M, U, and N constitute a computation graph characterized by dual inputs and dual outputs. In Step 3, P is be modelling to a series of convolutional operators with different kernel sizes. M, A, U, and N are implemented as linear transformations parameterized by trainable weight matrices *W* (Figure 1c). The mathematical details of this implementation are provided in Experimental Section Equations (11)–(16). Models formalized by Equations (11)–(14) are termed VCNU. The closed‐loop system formed by VCNU, including components A, M, and U, which enable cyclic information flow, is referred to as the PBM. Additionally, the model constituted by the VCNU and Equation (16) is referred to as the VCogM. The algorithmic flow of the VCogM is illustrated in Figure 1b and **Algorithm** **3**.

### 2.2 The Unbiased Mapping Algorithm

**Figure 3:**
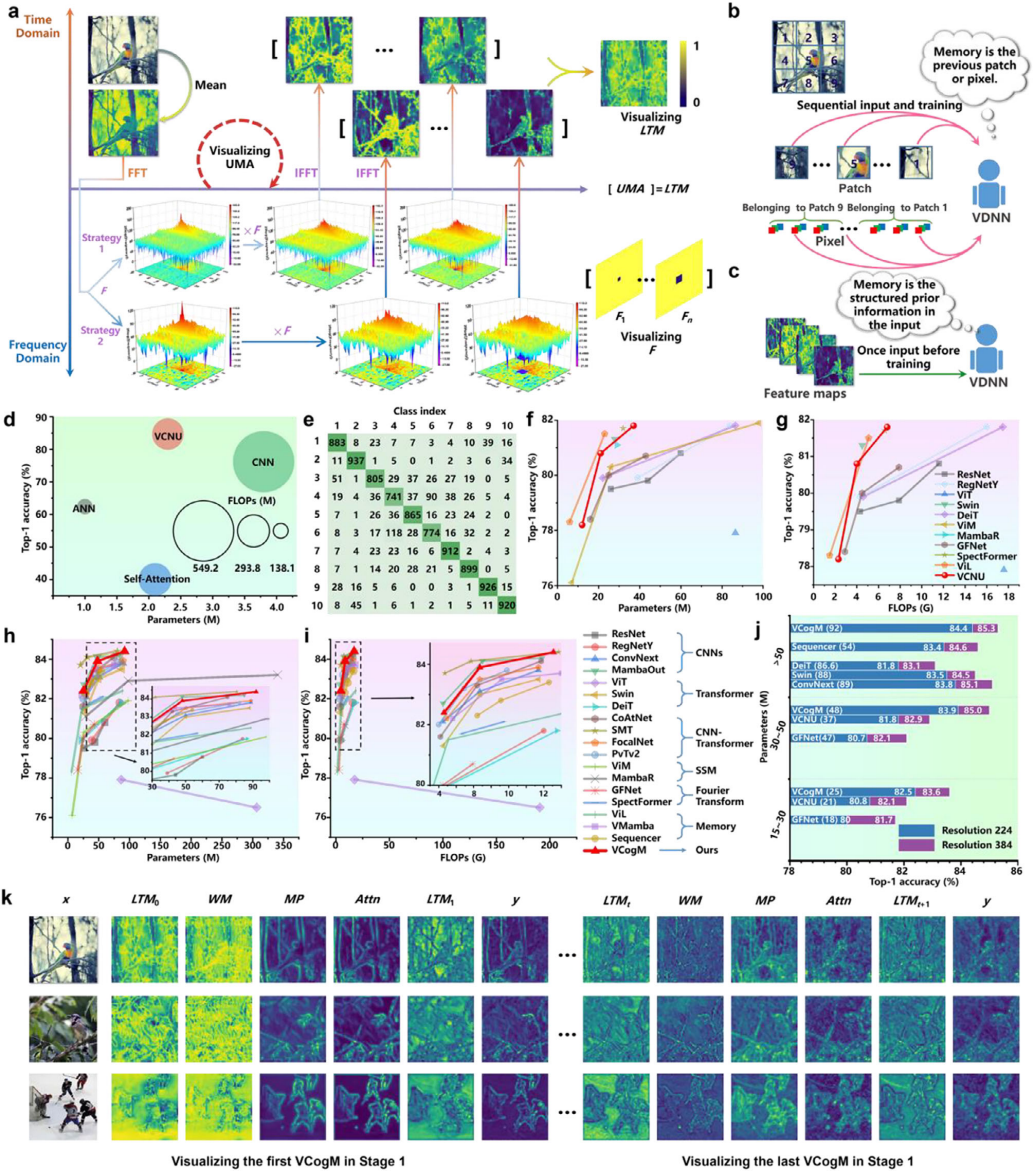
Experimental results of visual cognitive neural unit (VCNU) and visual cognitive model (VCogM) on natural scene recognition tasks. a) A visual overview of the UMA process. b) In CV, researchers commonly model memory by sequentially feeding serialized images into Visual Deep Neural Networks (VDNNs), using the previous patch or pixel as the network's memory. c) Proposed the memory of VDNNs. Inspired by the concept of visual templates, we define VDNNs' long‐term memory as prior information composed of basic image features. The UMA generates this prior information algorithmically. Compared to defining memory as pixel or patch, UMA‐generated feature maps carrying prior information are more cognitively plausible and provide higher‐level abstractions. d) Training results of VCNU versus classical operators in VDNNs on CIFAR‐10 dataset. e) Confusion matrix of VCNU on CIFAR‐10 test set. f,g) The performance of VCNU when scaled up and trained on the ImageNet1K large benchmark dataset. h,i) The performance of VCogM when scaled up and trained on the ImageNet1K. j) The results of pre‐trained at an input resolution of 224 × 224 and further fine‐tuned at a resolution of 384 × 384. k) Visualization of the feature maps in VCogM.

LTM is stored in the brain in a priori form independently of the task being learned and performed. To model LTM, we proposed a novel construction method called UMA (Figure 1d). The UMA explicitly utilizes the structural information of contour and texture features in an image as the core components of memory. UMA offers a robust framework for modeling structural information by integrating algorithmic and statistical components. The algorithmic component constructs a set of low‐pass filters *F* = {*F_i_* | *i* = 1, 2, …, *n*} with base *n* (Equation (2)), which modulate the spectrum of the input feature map in the frequency domain (Equation (1)). Leveraging the properties of the FFT and Inverse FFT (IFFT), low‐pass filtering in the frequency domain using *F* preserves contour features while blurring texture features, thereby capturing essential structural information for memory representation (**Figure** **3**a).

To recover contour features from blurred texture features, we employed the exponential power function *e^‐x^* to nonlinearly map the modulated feature maps (Equation (4)). Since *e^‐x^* nonlinearly transforms values from the input domain [0, +∞) to the range (0, 1], the UMA generates feature maps where contour positions are assigned a value of 1, aligning with the characteristics of a visual template (Figure 3a). Furthermore, the UMA operates independently of the model training process, derived solely through frequency domain operations. The statistical component provides a method for deriving the prior knowledge required by the UMA. Due to the conjugate symmetry of the spectrum obtained through FFT processing, the value of *n* is always finite. For the entire dataset, we analysed the *i*‐th sample by setting *n* to its maximum value and using the structural similarity (SSIM)^[^ ^48^ ^]^ to measure the quality of feature maps before and after the UMA transformation. To ensure that the UMA output retains the structured information from the input features, we imposed the constraint SSIM>0. By solving for this constraint (Equation (7)), we obtained the upper bound *N_i_* for *n* in the UMA. Extending Equation (7) to the entire dataset yields the statistic *N* = {*N_i_* | *i* = 1, 2, …, *β*}∈[0, *n_max_*]. We then applied frequency statistics to transform *N* into a frequency distribution *P*, as described in Equation (8). The *P* represents the statistical upper bounds on *n* across the dataset that satisfies the constraint.

The results of VCNU on ImageNet‐1K indicate that the VCNU achieves optimal performance when the truncated mean of *P*, after removing irrelevant data, is used as the solution. This finding is supported by Equation (9) and Table S4, Supporting Information. By solving for the parameter *n* using statistical methods, we introduced a priori information at the algorithmic level. Although this prior information is derived from the data itself, it functions similarly to traditional prior knowledge. Specifically, it enables the model to extract structured information from images before training begins (Figure 3c). The pseudocode of the UMA is illustrated in **Algorithm** **1** and **Algorithm** **2**.

Algorithm 1 **Unbiased Mapping Algorithm (UMA)**

**Table:** 

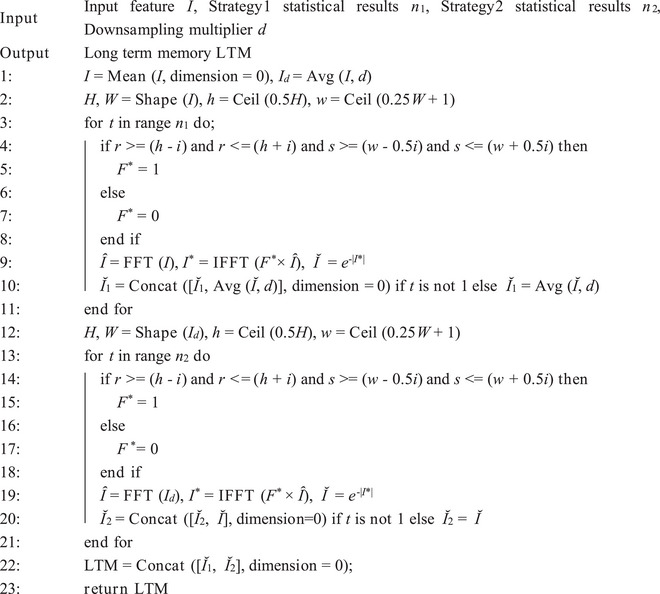

Algorithm 2 **The statistic algorithm of UMA**

**Table:** 

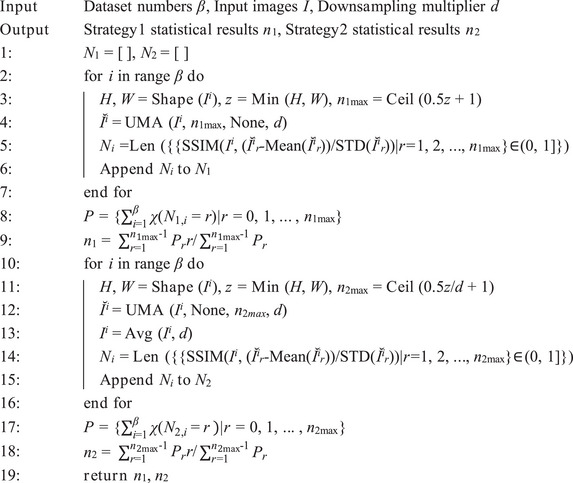

Algorithm 3 **Vision Cognitive Model (VCogM)**

**Table:** 

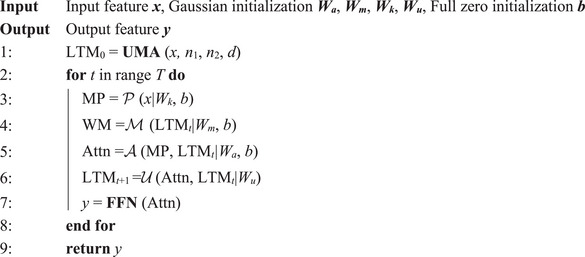

### 2.3 Scientific Validation Results and Analysis of CMF

VCNU and VCogM serve as fundamental components of the VDNN, enabling extension to larger‐scale networks through various connection architectures. We provided an example of scaling VCNU and VCogM to large‐scale VDNNs, with detailed descriptions in Table S8, Supporting Information. To validate the performance of VCNU and VCogM, we conducted experiments on three tasks: 1) the base operator validation task, 2) the visual recognition task for natural scenes, 3) the visual recognition task for agricultural objects.

#### Base Operator Validation Results and Analysis

We cascaded all the base operators into four groups to train on CIFAR‐10.^[^ ^49^ ^]^ VCNU achieved a Top‐1 Accuracy (ACC) of 84.7%, which represents state‐of‐the‐art (SOTA) performance (Figure 3d). Notably, VCNU has fewer parameters and lower computational complexity compared to the CNN, yet its Top‐1 ACC is 8.5% higher. Figure 3e demonstrates that VCNU effectively distinguishes most sample categories among the 10 target classes in CIFAR‐10.

For large‐scale testing on ImageNet‐1K,^[^ ^50^ ^]^ the results are shown in Figure 3f,g. VCNU‐21 M is outperformed by ResNet‐26 M,^[^ ^51^ ^]^ which consists of CNN operators, and it achieves a 0.9% higher Top‐1 ACC than the significantly larger RegNetY‐39 M.^[^ ^52^ ^]^ Moreover, VCNU‐37 M matches the Top‐1 ACC of RegNetY‐84 M. Additionally, VCNU‐37 M outperforms models such as Vim‐26 M,^[^ ^53^ ^]^ MambaR‐29 M,^[^ ^54^ ^]^ GFNet‐43 M,^[^ ^55^ ^]^ SepctFormer‐32 M,^[^ ^56^ ^]^ and CoAtNet‐25 M.^[^ ^57^ ^]^ The above results show that VCNU could be a base operator for VDNNs.

#### Benchmarking Results and Analysis for the Natural Scene Recognition Task

Figure 3h,i presents that VCogM‐48 M achieves a higher Top‐1 ACC than memory‐involved control models such as ViL‐89 M,^[^ ^35^ ^]^ Vamaba‐76 M,^[^ ^36^ ^]^ and Sequencer‐54 M.^[^ ^37^ ^]^ Furthermore, VCogM‐92 M achieves SOTA performance with a Top‐1 ACC of 84.4%. Compared to other models, VCogM demonstrates stronger scaling properties as the parameter size increases. Figure 3j illustrates the results of models pre‐trained at an input resolution of 224 × 224 and further fine‐tuned at a resolution of 384 × 384. VCNU and VCogM exhibit improved performance, with VCogM‐92 M achieving a Top‐1 ACC of 85.3%, representing SOTA performance. Although the computational complexity of VCogM‐92 M is 5.1 GFLOPs higher than that of DeiT‐86.6 M,^[^ ^58^ ^]^ its Top‐1 ACC is 2.2% better.

Figure 3k shows that the LTM constructed by the UMA appears blurred. In contrast, the visualization results of WM exhibit a more consistent color distribution concerning MP, Attn, and *y*, along with fewer shadows and clearer contours near the target. This suggests that the model can recall and reconstruct distinct features from blurred prior information, akin to how humans reconstruct clear WM from blurred LTM. In VCogM and VCNU, the PBM provides an input‐independent representation of features and establishes a pipeline for cyclic computation. VCogM actively encodes important features from Attn into LTM while forgetting irrelevant interference. As a result, the updated LTM (such as LTM_1_ and LTM_0_) displays clearer target profiles.

The MP results show that VCogM's multi‐scale perceptual capability effectively forms bottom‐up attention from the inputs. In the first stage of VCogM, LTM introduces some interference for Attn. However, compared to MP and WM, Attn's visualization results show a more accurate and clearer focus on the target's silhouette. This indicates that Attn is the outcome of filtering and coupling feature representations from WM and MP. Additionally, as the model depth increases, Attn reduces its focus on background regions, effectively separating the target from background features. The visualization results of VCogM demonstrate that CMF's cognitive functions are not executed independently but result from the coordinated execution of various functional functions driven by computational graphs. The experimental results provide strong evidence that the learning objective of VCogM is not merely to approximate the input‐output mapping function, but also to achieve coordinated optimization around sub‐functions such as multi‐scale perception, working memory generation, and attention formation.

#### Test results and Analysis of the Agricultural Object Recognition

**Figure 4:**
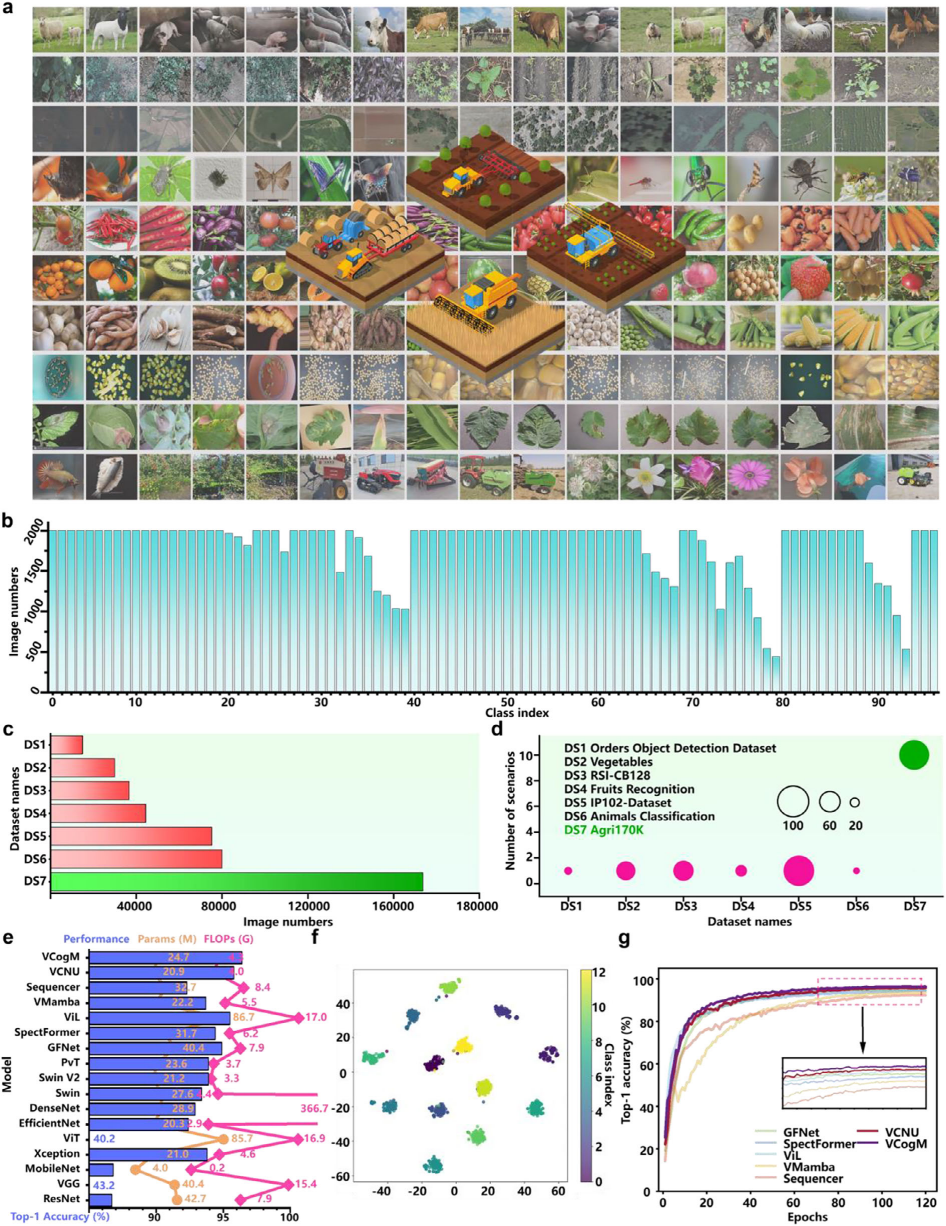
Experimental results of the Agri170K dataset. a) Sample images from the Agri170K. b) Illustration of the number of images per category in Agri170K. The actual meanings of the category indices are provided in the Table S3, Supporting Information. c) Comparison of data volume with common agricultural benchmark datasets. d) Comparison of scenarios covered by Agri170K and other common agricultural benchmark datasets. e) The results on Agri170K. VCogM achieves the best performance, with a Top‐1 Accuracy of 96.4%. f) t‐SNE visualization of VCogM. g) Training process visualization for all models on Agri170K.

**Figure** **4**c,d shows that developed Agri170K has significant advantages in terms of data capacity and agricultural scenario categories. Subsequently, we compared the performance of VCogM and VCNU with the top 10 most frequently cited models in agriculture, FFT‐based models, and memory models (Figure 4e). The results show that some models struggle to address agricultural image classification challenges, resulting in poor performance.

For instance, Sequencer‐38 M and ViL‐89 M achieve higher Top‐1 ACC than Swin‐28M^[^ ^59^ ^]^ on ImageNet‐1K, but the opposite is true on Agri170K. In contrast, VCogM‐24.7 M achieves SOTA Top‐1 ACC among all control models, while VCNU‐20.9 M ranks second, outperforming all other controls. The t‐SNE^[^ ^60^ ^]^ result further reveals that feature representations generated by VCogM for the same agricultural scenario are clustered together, while feature representations for different scenarios are well‐separated (Figure 4f). Moreover, as illustrated in Figure 4g, the training processes of VCogM and VCNU are more stable. These results indicate that the performance of VCogM and VCNU is robust and not dependent on specific data volumes or patterns. Experimental results across natural and agricultural scenarios demonstrate the strong generalization and learning capabilities of VCogM and VCNU for handling diverse features and data distributions, fully reflecting the rationality of cognitive heuristic modeling concepts.

### 2.4 The Advancement of Statistical Prior Knowledge

**Figure 5:**
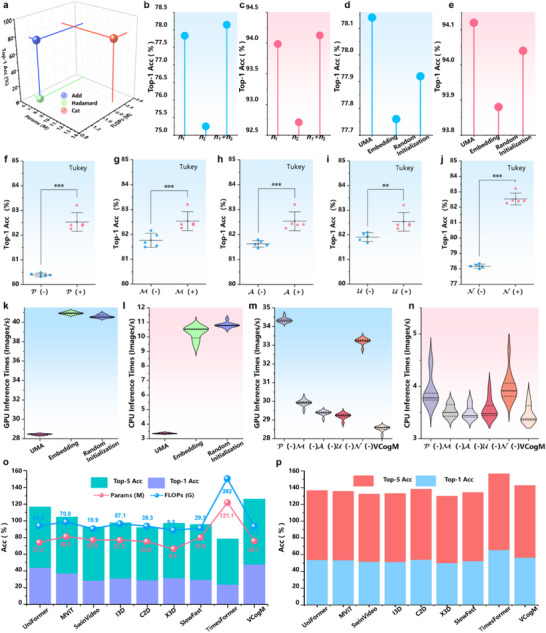
The results of ablation and cross‐domain validation experiment. a) The controlled experiment result of different aggregation methods in A of VCNU‐13 M. b,c) The controlled experiment result of feature map generation strategy in UMA within VCNU‐13 M. d,e) The controlled experiment result of UMA initialization and common parameter initialization methods for LTM of VCNU‐13 M. f,j) Univariate significance analysis of the effects of different modules on VCogM‐25 M performance. *** denotes *p* < 0.0001, ** denotes *p* < 0.01, * denotes *p* < 0.05. (+) denotes the module included in the model. (−) denotes the module not included in the model. k,l) Experimental results of different LTM initialization methods on inference speed of VCNU‐13 M. m,n) Experimental results of per modules on inference speed of VCogM‐25 M. All inference speeds were measured by calculating 100 images per run on a single RTX 4090 or Intel 8352 V, with the results averaged over 10 runs. o) The cross‐domain validation experiment results of VCogM using a training‐from‐scratch pipeline for action recognition task on HMDB51 dataset. p) The cross‐domain validation experiment results of VCogM using a transfer learning pipeline for action recognition task on HMDB51 dataset.

**Figure** **5**a–c and Tables S4–S7, Supporting Information show that multi‐scale perception does not necessarily improve with increased scales (Table S4, Supporting Information Step (a)), and the optimal configuration for P in VCNU is achieved with four parallel convolution operators. Additionally, VCNU performs best when the truncated mean of *P*—after filtering out irrelevant data—is used as *n* in the UMA statistical method. As shown in Figure 5d,e, the performance gain brought by UMA initialization is superior to that of embedding and random initialization methods. Considering cognitive rationality, UMA provides richer structured prior information than pixel representation, directly aligning with the concepts of LTM prior knowledge and memory templates and providing interpretable method support for memory modeling in VDNNs. Considering parameter optimization, the prior generated by UMA can provide better starting points and constraints for optimization. In addition, the UMA‐generated prior is generated based on probability distributions, which inevitably introduces additional noise and perturbations to the optimization process. These interferences force the model to rely on more abstract and stable feature representations, thereby learning more resilient and semantically grounded representations. From a cognitive perspective, this interference can be viewed as simulating the ambiguity mechanism of LTM.^[^ ^13^, ^18^ ^]^

Single‐factor ablation experiments showed that the VCogM, which integrates all modules, achieved the highest Top‐1 ACC of 82.5% (Figure 5f–j). The inclusion of modules A, P, M, and N in the VCogM model had a highly significant impact on the model's Top‐1 Accuracy (*p*‐value < 0.0001), while module U also had a significant impact on the VCogM model's performance (*p*‐value < 0.01). In summary, the five modules aligned with cognitive functions constitute the complete cognitive architecture of VCogM.

As observed in Figure 5k–n, the parallelization bottleneck in the FFT within the UMA is the primary factor contributing to the reduction in inference speed. It is important to clarify that the core objective of this study is to construct VDNNs aligned with human cognitive systems based on cognitive theory, thereby enhancing their predictive performance across various tasks. The FFT serves as the implementation method for constructing the LTM in VDNNs. Therefore, addressing the FFT's inherent parallelization computation bottlenecks is not within this study's primary scope. In the future, our primary research direction will be to develop lighter, more efficient cognitive‐inspired operators based on CMF.

We selected the video understanding task to validate the scalability and generalization of CMF and UMA across domains. In the video understanding domain, the input consists of a set of sampled frames (RGB) from a video. VCogM is not suitable for video analysis tasks. Therefore, we replaced the original 2D convolutions and regularization with 3D convolutions and regularization to adapt VCogM to video frame inputs and conducted comparative experiments with multiple expert models on the HMDB51^[^ ^61^ ^]^ dataset. Please refer to the supporting materials for details on experimental settings and modifications. As shown in Figure 5o,p, VCogM achieved the highest Top‐1 ACC of 48.04% and Top‐5 ACC of 78.04% in training from scratch and maintained competitiveness under fine‐tuning on ImageNet‐1K. The experimental results demonstrate that VCogM can be effectively extended to action recognition tasks, further validating the effectiveness of CMF.

### 2.5 The Flexibility and Scalability of CMF

**Figure 6:**
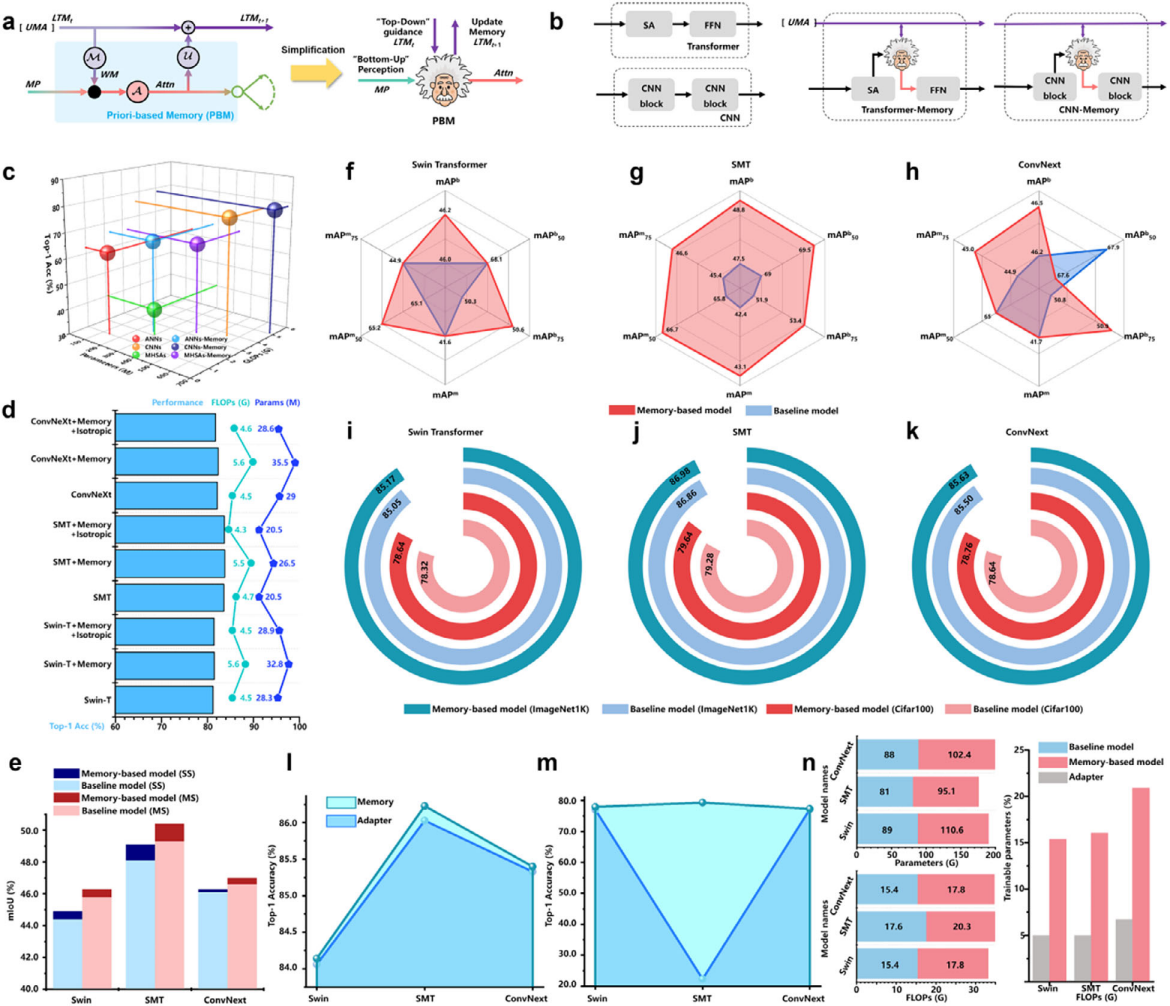
Illustration of the proposed prior‐based memory framework (PBM). a) Simplified illustration of PBM. b) An example of PBM embedded within Transformer and CNN architectures. PBM can flexibly combine with other operators, endowing existing operators or VDNNs with memory capabilities and making their attention mechanisms more cognitively plausible. c) The experimental results of PBM were embedded in CNN operators, artificial neural network (ANN) operators, and self‐attention operators on the CIFAR‐10 dataset. d) The performance of PBM when embedded in SwinTransformer, ConvNext, and SMT models on the ImageNet1K dataset. e) The results of PBM embedded in SwinTransformer, ConvNext, and SMT models were used as backbones for UperNet on the ADE20K semantic segmentation dataset. f) The results of PBM embedded in SwinTransformer, ConvNext, and SMT models were used as backbones for Mask RCNN on the COCO2017 object detection dataset. i,k) Full fine‐tuning (FT) results on ImageNet1K and CIFAR‐100. l,m) Parameter‐efficient fine‐tuning (PEFT) results on ImageNet‐1K and CIFAR‐100. For ImageNet‐1K fine‐tuning, all models use pre‐trained weights from ImageNet‐22K. For CIFAR‐100 fine‐tuning, all models use pre‐trained weights from ImageNet1K. n) Parameter and computational complexity changes.

CMF does not predefine a unique FF and its agency program, meaning an existing operator can replace any node in the computational graph. Considering that most existing VDNN operators do not involve memory concepts, we integrated PBM into representative baseline models, naming the resulting control groups with a suffix (such as Swin‐Memory). Baseline and control models are trained and validated on CIFAR‐10 and ImageNet‐1K (**Figure** **6**a,b). See Figure S2c, Supporting Information for integration guidelines.

On the CIFAR‐10, the control groups with PBM improve the Top‐1 ACC of the original baseline operators by an average of 10.4%, with SA‐Memory achieving a 25.3% improvement over the original baseline (Figure 6c). On the ImageNet‐1K, the control groups outperform Swin‐Transformer, ConvNext,^[^ ^62^ ^]^ and SMT^[^ ^4^ ^]^ by 0.28%, 0.24%, and 0.21%, respectively (Figure 6d). However, the integration of PBM introduces additional parameters and computational complexity. Even with similar parameter scales, all models with PBM outperform their original models (Figure 6d). This confirms that the closed‐loop cognitive system formed by memory and attention in PBM is the key to performance enhancement, rather than being caused by parameter scale expansion.

Figure 6e–h shows the experimental results of all control groups as backbones for UperNet^[^ ^63^ ^]^ and Mask‐RCNN^[^ ^64^ ^]^ on ADE20K^[^ ^65^ ^]^ and COCO2017,^[^ ^66^ ^]^ respectively. In COCO2017, mAP^b^ and mAP^m^ improved by an average of 0.6% and 0.23%, respectively. In ADE20K, MIoU (SS) and MIoU (MS) improved by an average of 0.57% and 0.69%, respectively. Figure 6d–h illustrates that PBM can be flexibly combined with other operators or VDNN models, showcasing CMF's broad applicability.

### 2.6 The Framework of Prior‐Based Memory in Transfer Learning

We tested the performance of PBM using full fine‐tuning (FT) and parameter‐efficient fine‐tuning (PEFT). Figure 6i–k presents introducing PBM into the baseline model improved the Top‐1 ACC by an average of 0.12% on ImageNet‐1K and 0.2% on CIFAR‐100.^[^ ^49^ ^]^ These results demonstrate that PBM effectively enhances knowledge transfer quality across large and small datasets, regardless of the original model architecture.

As shown in Figure 6l, m, compared to the Adapter,^[^ ^67^ ^]^ the baseline model with PBM integrated improves the Top‐1 ACC by an average of 0.11% on ImageNet‐1K and 58.03% on CIFAR‐100. Notably, SMT‐Adapter experiences training crashes on the CIFAR‐100, while PBM, as a PEFT method, delivers more stable training results across the three architectural models. This indicates that PBM can serve as a plug‐in module to quickly equip any VDNN with memory capabilities, making it highly effective for transfer learning applications. Since PBM is not specifically designed for transfer learning tasks, introducing PBM into FT and PEFT will result in additional parameters and computational complexity (Figure 6n and Table S12, Supporting Information).

Overall, PBM complements the memory mechanism of the baseline model and demonstrates its effectiveness in diverse image processing tasks and transfer learning tasks. By introducing a top‐down memory‐driven mechanism, PBM provides the model with explicit inductive biases and semantic priors, constraining the model to follow computational paths consistent with human cognitive patterns to recall, modify, and reconstruct domain knowledge, thereby improving the quality and stability of knowledge learning.

## 3 Conclusion

We introduce a three‐stage VDNN theoretical framework (CMF) comprising functional abstraction, operator structuring, and program agent designed to translate highly abstract cognitive theories into mathematical expressions and trainable operators. CMF introduces three core concepts: FF, computation graph, and agency program, which allow cognitive functions and the causal logic between them to be embedded in the computational process of VDNNs, thereby becoming explicit constraints on the computational principles and learning objectives of operators.

To address the gap in VDNNs’ memory concept, this study proposes UMA, which defines VDNNs’ LTM as prior information composed of basic features in images and uses FFT and statistical methods to unsupervised extract these basic features from images. Compared to treating serialized pixels as LTM, UMA provides richer feature representations by highlighting higher‐level abstractions such as contours and textures. More importantly, UMA offers an LTM modeling method for VDNNs that aligns with cognitive science concepts. CMF and UMA lay the theoretical foundation and implementation path for constructing cognitively inspired VDNNs.

CMF bridges the representation gap and conceptual gap between cognitive theory and VDNNs. The former refers to the difficulty of mathematically formalizing theoretical concepts, while the latter reflects the challenge of mapping theoretical concepts into scalable training agents. More importantly, CMF uses cognitive functions as the basis for model design and cognitive logic as topological constraints for structure, thereby constructing operators and VDNNs that more closely resemble the operational mechanisms of human intelligence. This paradigm holds promise for advancing the application of cognitive theories in artificial intelligence. Additionally, CMF allows replication of brain‐like cognitive mechanisms within VDNNs, providing a promising pathway for cognitive psychologists to leverage numerical simulation in quantifying and exploring the cognitive processes of the human brain.

## 4 Experimental Section

### Unbiased Mapping Algorithm

Memory is the brain's retention of past experiences and knowledge, providing additional cognitive representations. The problem addressed was creating a memory for VDNNs in image processing tasks that lack temporal characteristics. To solve this, this work proposed the UMA, a method for constructing LTM based on prior information. UMA consists of algorithmic and statistical components.

**Related concepts**. The filtering algorithm was an effective feature extraction method that retains images’ contour or texture information of images without requiring training. The premise of this approach was constructing of a deterministic filter F^*^. Assuming F^*^ is known, the frequency‐domain filtering algorithm can be expressed as:

(1) $$I^{*}=\mathrm{IFFT}\left(\underset{=⌢I}{\underset{︸}{\mathrm{FFT}\left(I\right)}}\cdot F^{*}\right),\;⌢I,\;F^{*}\in R^{H\;\times \left\lceil \frac{W}{2}+1\right\rceil }$$

where *I* denotes the input image with dimensions *H* × *W*; ⌢I denotes the spectrum of *I*; and I^*^ denotes the spectrum of *I*.

Assume a set of filters *F* with a cardinal number of *n* exists. Substituting each element of *F* into Equation (1) yields a series of feature maps *I_m_* that contain contour features. When *F* depends on the statistical results from a specified dataset, the above method satisfies the definition of LTM in VDNNs. However, this assumption presents three challenges: (1) How to model a set of feature maps *I_m_* that include contour and texture features. (2) How to construct *F* using statistical methods. (3) How to quantify the quality of images before and after filtering, which is a prerequisite for performing statistics.

**UMA's algorithmic component**. The UMA partially addresses the first challenge, and the specific algorithm flow is as follows. Consider a set of filters *F* = {*F_i_* ∈ℝ*^H^* ^×^ *^W^ |i* = 1, 2,…, *n*}, where each *F_i_* satisfies the following definition:

(2) $$F_{i}=\left\{\begin{array}{c} 1,\;\mathrm{if}\;\left[h-L\left(i\right),w-\left\lfloor \frac{L\left(i\right)}{2}\right\rfloor \right]\leq \left(r,\;s\right)\leq \left[h+L\left(i\right),\;w+\left\lceil \frac{L\left(i\right)}{2}\right\rceil \right]\\ 0,\;\mathrm{otherwise} \end{array}\right.,\;h=\left\lceil \frac{H}{2}\right\rceil ,\;w=\left\lceil \frac{W}{4}+1\right\rceil$$

(3) L(i)=i

where *r* represents the horizontal coordinate in the frequency domain; *s* represents the vertical coordinate in the frequency domain; *F_i_* is an ideal low‐pass filter with a rectangular shape, where the central region is entirely zero; and *L* is the filter construction function that determines the filtering range of *F_i_*.

If *F* is used to modulate the spectrum ⌢I, it will produce a set of filtered images *I_m_* = {I_i_^*^ ∈ℝ*^H^* ^×^ *^W^ |i* = 1, …, *n*}. Due to the time‐frequency invariance of FFT and IFFT, if *I*∈[−1,1], then I_i_^*^ ∈[−1,1], and the contour position values are zero. To reconstruct the contour features from I_i_^*^, a negative exponential function of *e* is introduced to perform a nonlinear mapping of |I_i_^*^|. This process is defined as:

(4) $$⌣I=\left\{e^{-\left|I_{i}^{*}\right|}\in 0,\;1|\;i=1,\;2,\;\cdots ,\;n\right\}=\mathrm{UMA}\left(I,\;n\right)\in \left[0,1\right]$$

where *Ĭ* represents the output of the UMA. The negative exponential function of *e* assigns values of 1 to the contour positions in I_i_^*^ and applies a nonlinear decay to other positions. To enhance the model's robustness to color, when *I* is an RGB image (*I*∈ℝ^3 ×^ *^H^* ^×^ *^W^*), the image *I* is converted to grayscale before applying the UMA.

**UMA's statistical component**. The statistical part of the UMA addressed the latter two challenges. This work's goal is to make *F* statistically representative across the entire dataset. From Equation (2), it is known that *F* depends on *n*. Therefore, *n* is considered a statistic. Given that the spectrum ⌢I after FFT exhibits conjugate symmetry, the range of *n* is defined as *n*∈[0, ⌈0.5min(*H*, *W*)⌉+1]. This indicates that *F* is always finite. First, this work considered constructing *F* using *n*_max_. This work treated the target dataset as the statistical population and substitute the *i*‐th sample *I^i^* into Equation (4) to obtain the set *Ĭ^i^*. To measure the quality of the UMA output, this work used SSIM as an intermediate metric to quantify the relationship between each element in *Ĭ^i^* and the *I^i^*. SSIM is commonly used to measure the structural similarity between images and is defined as:

(5) $$\mathrm{SSIM}\left(p,\;q\right)=\left[L\left(p,\;q\right)\times \;C\left(p,\;q\right)\times \;S\left(p,\;q\right)\right]\in \left[-1,1\right]$$

where *L* represents luminance contrast, *C* represents the contrast, *S* represents the structure; *p*, *q* are images.

Considering the value ranges of *Ĭ^i^* and *I^i^*, this work first applied standard normalization to each element in the set *Ĭ^i^*. Then, this work computed the corresponding SSIM values between the normalized elements and *I^i^*. The computation formula is as follows:

(6) $$\begin{array}{c} U^{i}=\left\{U_{r}^{i}=\mathrm{SSIM}\left(I,\;\frac{⌣I_{r}^{\;i}-\mathrm{Mean}\left(⌣I_{r}^{\;i}\right)}{\mathrm{STD}\left(⌣I_{r}^{\;i}\right)}\right)|r=1,\;2,\;\cdots ,\;n_{\max}\right\}\in \left[-1,1\right] \end{array}$$

where *U^i^* denotes the set of SSIM values between the output of the UMA for the *i*‐th sample in the statistical population at *n* = *n_max_* and that sample. Given the properties of the SSIM value range, this work used *U^i^* ∈(0,1] as a constraint to obtain the subset *U*_+_ *^i^*, which is defined as:

(7) $$U_{+}^{i}=\left\{U^{i}\in \left(0,\;1\right]\right\}$$

The cardinal number *N_i_* ∈[0, *n_max_*] of *U*_+_ *^i^* represents the number of feature maps for the *i*‐th sample in the population that exhibits structural similarity before and after UMA transformation. If *N_i_* ≠ 0, there exists an *α*∈(0, *N_i_*] such that *U^i^* |*_n = α_* ∈(0, 1], where *U^i^* |*_n = α_* denotes the set of SSIM values between the feature maps before and after UMA processing for the *i*‐th sample at *n* = *α*. Therefore, *N_i_* reflects the upper bound of *n* in the UMA, which satisfies the constraint for a given sample. To generalize Equation (7) from a single sample to the entire population (with size *β*), we obtained a set *N* composed of *N_i_*, that is, *N* = {*N_i_* | *i* = 1, 2, …, *β*}∈[0, *n_max_*]. The set *N* represents the collection of upper bounds of *n* in the UMA that satisfy the constraint across all samples. To further quantify this, this work used frequency statistics to convert *N* into a frequency distribution, denoted as the set *P*. The *P* indicates the number of occurrences of each element in *N*, representing the statistical results of the upper bounds of *n* that satisfy the constraints across the population. The calculation process is as follows:

(8) $$P=\{P_{r}=\sum_{i=1}^{\beta }\chi \left(N_{i}=r\right)|\;r=0,\;1,\;\cdots ,\;n_{\max}\}$$

where χ denotes the indicator function, which is used to determine whether *N_i_* is equal to *r*; *r* represents a possible value, and *P_r_* denotes the corresponding frequency.

Selecting appropriate statistics such as the mean, truncated mean, or median allows us to determine the value of *n*. Further, *r* = 0 indicates that any cutoff frequency will result in UMA failure, that is., ∀*r*∈[1, *n*_max_], *U^i^* |*_n = r_* ∉ (0, 1]. *r* = *n*_max_ indicates that any cutoff frequency satisfies the UMA design requirements, that is., ∀*r*∈[1, *n*_max_], *U^i^* |*_n = r_* ∈(0, 1]. Therefore, when *r* = 0 or *r = n*_max_, *P_r_* can be considered as invalid data. Thus, the solution for *n* can be defined as the truncated mean of *P* after removing the irrelevant data, as defined by:

(9) $$n=\frac{\sum_{r=1}^{n_{max}-1}P_{r}r}{\sum_{r=1}^{n_{max}-1}P_{r}}$$

To enhance the diversity of UMA outputs and introduce a rich variety of prior information into the memory of VDNNs, this work defined two LTM modeling strategies based on UMA. Assume the downsampling factor is *d*. Strategy 1 is designed to execute the UMA and then perform *d*‐fold downsampling. Strategy 2 is designed to perform *d*‐fold downsampling and then execute the UMA. Finally, the outputs from both strategies are concatenated to form the LTM for VDNNs. Additionally, to fully leverage the acceleration benefits of GPU parallel computing, the outputs *Ĭ* are concatenated together. The initial LTM is defined as:

(10) $$\begin{array}{c} \left\{\begin{array}{c} \mathrm{Strategy}1:⌣I_{1}=\mathrm{Avg}\left(\mathrm{UMA}\left[I;\;n_{1}\right];\;d\right)\in R^{n_{1}\times \frac{H}{d}\times \frac{W}{d}}\\ \mathrm{Strategy}2:⌣I_{2}=\mathrm{UMA}\left[\mathrm{Avg}\left(I;\;d\right);\;n_{2}\right]\in R^{n_{2}\times \frac{H}{d}\times \frac{W}{d}} \end{array}\right.\\ \mathrm{LT}M_{0}=\mathrm{Concat}\left(⌣I_{1},\;⌣I_{2}\right)\in R^{\left(n_{1}+n_{2}\right)\times \frac{H}{d}\times \frac{W}{d}} \end{array}$$

where Avg denotes average downsampling; LTM_0_ represents the initial long‐term memory; *n*_1_ and *n*_2_ represent the values of *n* corresponding to Strategy 1 and Strategy 2, respectively, as determined by Equation (9). The algorithmic part of the UMA is implemented in Algorithm 1, while the statistical part is implemented in Algorithm 2. The results of UMA in all datasets are implemented in Table S1, Supporting Information.

### The Design Principle of VCNU and VCogM

This section primarily introduces the computational representations and algorithmic formulations of each FF in **Step 3**. The P is to simulate the multi‐scale perception process of bottom‐up attention formation in the human brain, to extract and select diverse feature representations from images. CNNs use convolution operators with a kernel size of *k* × *k* to perform weighted averages over sliding windows, mimicking the limited receptive fields of neural cells. Based on this, P is designed as *j* parallel convolution operators, each with a convolution kernel size of *k* × *k*, where *k* = 2*i*+1. To reduce the computational complexity, the input *x* is divided along the channel dimension *D* into *j* parts, that is., *x =* {*x_i_*, *i =* 1, 2, …, *j*}. Each of the *j* convolution operators processes its corresponding subset *x_i_* independently. Finally, the results from all convolution operations are concatenated to form MP. P can be represented as:

(11) $$\begin{array}{cc} \mathrm{MP} & =P\left(x\right)=\sum_{i=1}^{j}\mathrm{Concat}\underset{\mathrm{convolution}\;\mathrm{operation}}{\underset{︸}{\left(x_{i}*W_{k}\right)}}\\ & \;+b,x_{i}\in R^{\frac{D}{j}\times \;H\;\times \;W},\;k=2i+1 \end{array}$$

where *W_k_* denotes the weight matrix of convolution kernel size of *k* × *k*, *W_k_* ∈ℝ*^k^* ^×^ *^k^*.

The M is to simulate the top‐down attention process in the human brain, where prior representations stored in LTM are retrieved and reconstructed to form WM and create visual templates. The goal is to provide a priori feature representation independent of *x* based on LTM. According to the EPT, WM results from selective activation from LTM. Compared to LTM, WM should include more explicit feature representations relevant to the current task. Therefore, M is designed to use a linear mapping with parameters *W_m_* ∈ℝ *^D^* ^×^ *^n^* to reconstruct WM from LTM, where LTM_t_∈ℝ*^n^* ^×^ *^H^* *^W^* and the resulting WM∈ℝ*^D^* ^×^ *^H^* *^W^*, with *n* < *D*. M can be represented as:

(12) $$\mathrm{WM}=M\left(\mathrm{LT}M_{t}\right)=\underset{\mathrm{linear}\;\mathrm{projection}}{\underset{︸}{W_{m}\times \mathrm{LT}M_{t}}}+b\in R^{D\;\times \;\mathrm{HW}}$$

The A simulates the BCT's coupling process (including competition and bias) of two non‐homologous attention mechanisms. This work decomposed A into selection (activation) and integration (weighting). This work used an activation function Act to simulate feature retention and filtering and a linear mapping parameterized by *W_a_* to dynamically weight the selected features. The backpropagation algorithm, *W_a_* optimizes based on gradients, thereby simulating the dynamic coupling process between MP and WM. Since MP and WM have different source feature representations, to align them while effectively retaining their respective features, we concatenated MP and WM along the channel dimension *D* and applied data normalization Norm before passing them through the activation function. It should be noted that the dimension of MP is first transformed into ℝ^*D* × *HW*^. A can be represented as:

(13) $$\begin{array}{cc} \mathrm{Attn} & =A\left(M\left(\mathrm{LT}M_{t}\right),\;P\left(x\right)\right)\\ & =W_{a}\times \;\mathrm{Act}\left(\mathrm{Norm}\left(\underset{\mathrm{concat}}{\underset{︸}{\left[\mathrm{WM},\mathrm{MP}\right]}}\right)\right)+b\in R^{D\;\times \;\mathrm{HW}} \end{array}$$

where *W_a_* ∈ℝ *^D^* ^× 2^ *^D^*, Act denotes the GeLU activation function and Norm denotes the BatchNorm regularization function.

Research in cognitive science on EPT has shown that sustained attention to a target enhances the encoding and storage quality of that target in LTM, thereby increasing the likelihood of subsequent retrieval and recall.^[^ ^13^, ^18^, ^23^ ^]^ The U is designed to encode attention‐biased feature representations into LTM. U is designed to use a linear mapping parameterized by *W_u_* ∈ℝ^*n* × *D*^ to encode *Attn*∈ℝ*^D^* ^×^ *^HW^* into LTM∈ℝ*^n^* ^×^ *^HW^*. Since *D*>*n*, encoding Attn into LTM is essentially one of compression and abstraction, similar to the encoding process in human memory. Given LTM updates' slow and potentially forgetful nature, this work introduced additive operations and activation functions. First, this work added the newly encoded features from Attn to the current LTM_t_, which limits the update rate of LTM. Then, following the principles of LSTM, this work used an activation function to simulate forgetting. U can be represented as:

(14) $$\mathrm{LT}M_{t+1}=U\left(\mathrm{Attn},\;\;\mathrm{LT}M_{t}\right)=\mathrm{Act}\left(W_{u}\times \mathrm{Attn}+\mathrm{LT}M_{t}\right)\in R^{n\;\times \;\mathrm{HW}}$$

The N is to perform nonlinear feature extraction on attention‐biased targets, enabling the model to learn more complex relationships. In this study, N is directly replaced by an FFN, which can be expressed as:

(15) $$y=N\left(\mathrm{Attn}\right)=\mathrm{FFN}\left(\mathrm{Attn}\right)\in R^{D\;\times \;\mathrm{HW}}$$

### Training Methods and Loss Function

This work implemented all models using the PyTorch and MMCV frameworks. The hardware configuration included a 1T SSD, two AMD EPYC 7T83 CPUs, eight NVIDIA A100‐PCIE‐40GB GPUs, and 233 GB of RAM. The software environment consisted of Python 3.8.10, Ubuntu 18.04, CUDA 11.0, PyTorch 1.10.0, torchvision 0.11.0, and MMCV 2.1.0 for experiments.

To jointly optimize attention accuracy and memory effectiveness, we defined a composite loss function, which can be expressed as:

(16) $$\mathrm{Loss}\left(\hat{y};\;y_{x},\;y_{m}\right)=-\sum_{i=1}^{C}y_{x,\;i}\log\left({\hat{y}}_{i}\right)-0.5\sum_{i=1}^{C}y_{m,\;i}\log\left({\hat{y}}_{i}\right)$$

where $\hat{y}\in R^{C}$ denotes the predicted class probabilities over *C* categories, *y_x_* ∈ {0,  1}^*C*^ is the one‐hot encoded ground‐truth label vector, and *y_m_* ∈ {0,  1}^*C*^ represents a memory‐derived one‐hot encoded ground‐truth label vector.

The first part of the loss function is cross‐entropy loss,^[^ ^68^ ^]^ which optimizes the model's overall cognitive ability. The second part introduces auxiliary supervision from the memory mechanism, encouraging the model to align LTM with the attention target spontaneously. Tables S8 and S9, Supporting Information provide guidelines for integrating PBM into mainstream architectures, hyperparameters, and training settings for VCNU and VCogM when extended to large models.

To improve the model's robustness, the models follow the experimental settings of Swin Transformer and introduce techniques such as random resized crop, random horizontal flip, color jitter, and augmentation to the training data during the training stage. In video analysis tasks, the models follow the experimental settings of UniFormer^[^ ^69^ ^]^ and perform operations such as temporal clip sampling, scale jittering, random spatial crop, and random horizontal flip on the video during the training stage.

### Statistical Analysis

**Agri170K dataset**. Agriculture was a foundational industry in human society, encompassing numerous scenarios, including cultivation, livestock farming, machinery, breeding, and harvesting. This work's objective was to develop a high‐quality agricultural image dataset that covers different agricultural scenarios to validate the proposed methods, and the details are as follows.

**(1) Candidate Scenarios**. This work used “Agriculture Computer Vision” as the keyword to search for 244 relevant articles in the ScienceDirect, Springer, Wiley, and IEEE Xplore databases over the past 3 years. Table S2, Supporting Information presents these 13 agricultural scenarios and their frequency of application. This work selected the top 10 scenarios for Agri170K including animals, weeds, remote sensing, insects, fruits, vegetables, crops, crop seeds, pests and diseases, and agricultural machinery.

**(2) Data collection**. First, this work searched platforms such as Kaggle, HuggingFace, and ModelScope for all datasets related to our candidate scenarios. This search yielded 154 public datasets with over 2.26 million images. Second, this work designed multiple keyword combinations, including crops, pests, diseases, and farmland images to crawl various online sources. Sampling strategies were implemented to enhance data balance and quality, collecting more than 400 000 images. For open‐field targets such as remote sensing and weeds, this work used the DJI Air 2S equipped with high‐resolution cameras. For non‐open environments, such as crop seeds, we utilized the MV‐GE300C‐T industrial camera. From 2020 to 2024, this work conducted image collection in Yukou Town, Beijing (40°10′5″ N, 116°58′38″ E), Wulian County, Shandong Province, China (35°35′50″ N, 119°33′23″ E), Heluo Town, Henan Province, China (34°50′57″ N, 113°0′18″ E), and other places. These efforts resulted in the collection of 200 000 images. Combining these three data collection methods, this work gathered over 2.86 million agricultural images. These images serve as the raw data for creating the Agri170K dataset.

**(3) Data screening**. Initially, this work used the Pillow and OpenCV libraries to perform a preliminary screening of the raw image dataset. Then, to further reduce data redundancy, this work applied the data deduplication method outlined in,^[^ ^70^ ^]^ removing about 220 000 unparseable and 2.1 million redundant images. Subsequently, this work recruited 20 graduate students with agricultural backgrounds as screening personnel and two associate professors as inspection experts. Ultimately, this work obtained 430 000 high‐quality images.

**(4) Manual annotation**. Given the wide variety of agricultural target categories and the need for strong professional knowledge to distinguish between them, this work recruited six doctoral students with backgrounds in agronomy, crop science, and agricultural machinery, along with six associate professors, to classify and verify the images. To ensure category balance within the dataset and diversity within each category, this work conducted a secondary screening process during data classification. Ultimately, this work compiled an agricultural dataset, Agri170K, consisting of 96 categories and 173 691 high‐quality agricultural images (Figure 4a,b). From each of the 96 categories, this work randomly selected 100 images to form a validation set. In contrast, the remaining images constitute the training set.

**Statistical methods**. To test the impact of different modules on VCogM‐25 M performance and the necessity of each module, this work conducted five single‐factor experiments on the ImageNet‐1K dataset with five modules as independent variables. Each experiment was repeated five times, and Top‐1 ACC was used as the dependent variable. This work used Origin software to perform analysis of variance and Tukey's test on the results of the five experiments. All tests were conducted as two‐sided with a significance threshold set at a *p*‐value of <0.05.

### Description of Experiments and Details

To validate the proposed CMF and UMA, this work designed experiments in four parts: 1) This work compared VCNU and VCogM with the most representative operators and VDNNs on public benchmark datasets to verify the superiority of CMF and UMA. 2) This work evaluated methods in agriculture to demonstrate their generalization and societal value in real‐world scenarios. 3) This work presented a comprehensive ablation study. 4) To verify the scalability of the method, this work extended it to video recognition tasks. 5) This work assessed UMA and PBM using training‐from‐scratch and transfer learning methods on different tasks to highlight the design and performance. Detailed experimental setups and additional specifics are provided in Supporting Information.

## Conflict of Interest

The authors declare no conflict of interest.

## Author Contributions

G.L. and L.L. contributed equally to this work. G.L. and L.L. carried out the concept and designed the experiments. Y.D. and Z.S. supervised the investigations. G.L., L.L., X.L., and X.W. performed the experiments. X.L. and X.W. analyzed the data. G.L., L.L., and Y.D. wrote the paper. All authors have read and agreed to the published version of the manuscript.

The CIFAR‐10 and CIFAR‐100 datasets are available at: CIFAR‐10 and CIFAR‐100 datasets (toronto.edu). The ImageNet‐1k dataset is available at: https://www.image‐net.org/download.php. The COCO2017 dataset is available at: https://cocodataset.org/#download. The ADE20K dataset is available at: http://groups.csail.mit.edu/vision/datasets/ADE20K/. The HMDB51 dataset is available at: https://serre‐lab.clps.brown.edu/resource/hmdb‐a‐large‐human‐motion‐database/. The Agri170K dataset is available at: https://github.com/CAU‐COE‐VEICLab/Vision‐Cognitive‐Neural‐Networks. All data used in this paper are publicly available at https://github.com/CAU‐COE‐VEICLab/Vision‐Cognitive‐Neural‐Networks.

## Supporting information



Supporting Information

## Data Availability

All data used in this paper are publicly available and can be accessed at the following link after the paper is published. https://github.com/CAU‐COE‐VEICLab/Vision‐Cognitive‐Neural‐Networks. Additional codes supporting the results of this study are available from the corresponding author upon reasonable request.
