# Supplementary material for: The Potential of Cognitive‐Inspired Neural Network Modeling Framework for Computer Vision
Source: Adv Sci (Weinh). 2025 Aug 19;12(41):e07730. doi: 10.1002/advs.202507730 (PMC12591195; doi:10.1002/advs.202507730)
Supplement: Supplementary file 1 — Supporting Information [file ADVS-12-e07730-s001.docx]

**Supporting Information**

**The Potential of Cognitive-Inspired Neural Network Modeling Framework for Computer Vision**

*Guorun Li, Lei Liu, Xiaoyu Li, Yuefeng Du***, Zhenghe Song, Xiuheng Wu*

G. Li, L. Liu, X. Li, Y. Du, Z. Song, X. Wu

Detailed experimental setup are as follows.

**Part one**. In the first part, we select typical operators that constitute VDNNs as the control group. These include CNN operators, Linear Mapping (ANN), and Self-Attention mechanisms. These are compared with VCNU on the CIFAR-10 dataset. All models consist of four cascaded layers, each with identical input dimensions: [64, 128, 256, 512]. Detailed experimental procedures are provided in Table S9.

Additionally, we scale VCNU and VCogM to different parameter sizes according to the configurations in Table [S8](#TS8) (less than 10M, 20-30M, 30-50M, and greater than 50M). We then evaluate VCNU and VCogM quantitatively against other control models on the ImageNet1K public dataset. The selected control models include all memory-related models (ViL, VMamba, and Sequencer), FFT-related models (GFNet, SpectFormer), advanced CNN architectures, Transformer architectures, hybrid CNN-Transformer architectures, and Mamba architecture models, totaling 18 control models. Training details for all models follow in Table [S9](#TS9). The selected control models cover all types of VDNN architectures. It is important to note that while VCNU and VCogM do not involve FFT and only use UMA to initialize LTM, we still include FFT-based models in the control group to comprehensively evaluate the superiority of our proposed methods.

**Part Two**. We select commonly used VDNNs in agriculture, including ResNet, VGG, MobileNet, Xception, EfficientNet, DenseNet, Vision Transformer (ViT), Swin Transformer (Swin), Swin Transformer V2 (SwinV2), and Pyramid Vision Transformer (PvT). Additionally, all memory models and FFT models from **Part One** serve as the control group. All models are trained on the Agri170K dataset, with training details following in Table [S9](#TS9).

**Part Three**. For clarity, we divide the ablation experiments into eight steps, labeled **Step (a)** through **Step (h)**.

**Step (a)**. In the algorithmization of P, we employ *j* parallel convolution operators with varying kernel sizes. To investigate the optimal value of *j*, we use an incrementing sequence starting from 1 and assign these values to *j*, training the models on the ImageNet1K dataset.

**Step (b)**. In VCNU, we use concatenation to aggregate *WM* and *MP*. However, aggregation can also be achieved using Hadamard product and addition. Therefore, we conduct controlled experiments comparing these three aggregation methods as a single variable.

**Step (c)**. Since VCNU is designed as a hierarchical architecture, each stage requires down-sampling, which also applies to *LTM*. In VDNNs, down-sampling methods include average pooling (Avg), max pooling (Max), and convolutional down-sampling (Conv). We perform controlled experiments comparing these three down-sampling operations as a single variable.

**Step (d)**. In UMA, appropriate statistical measures such as mean, median, and trimmed mean are used to solve for *n*. To explore the impact of different statistical measures on VCNU, we select mean, median, trimmed mean, and trimmed median as the statistical measures for solving *n* and conduct controlled experiments.

**Step (e)**. We define two strategies within UMA. In this experiment, these two strategies are compared as a single variable.

**Step (f)**. In UMA, *L* can be defined not only as a linear function but also as other functions such as Fibonacci functions and piecewise functions. Therefore, we choose linear, Fibonacci, and piecewise functions as single variables for controlled experiments.

**Step (g)**. We select common parameter initialization methods for VDNNs, specifically embedding and random initialization, to initialize *LTM*. This comparison aims to explore the advantages of cognitively plausible memory modeling methods.

**Step (h)**. To study the impact of each component in VCogM on its accuracy and reasoning speed, we use VCogM-25M as the baseline and conduct single-factor ablation experiments by sequentially removing P, M, A, U, and N.

**Part Four**. CMF and UMA focus on traditional image recognition tasks. However, to verify the applicability of the proposed method in different domains, we apply VCogM-25M to the action recognition task in the video understanding domain. Unlike **Part One** and **Part Two**, the input to the model in this task is no longer a single RGB image but a collection of sampled frames (RGB) from the video. To enable VCogM to be trained for this task, we replace the original 2D convolution with 3D convolution (frame is 1) and the original 2D BatchNorm with 3D BatchNorm, while keeping all other parameters and structures unchanged. This is a simple and widely adopted technique for transitioning from image to video domains, and it allows for weight sharing from image recognition tasks. We select nine typical video understanding models, including CNNs (SlowFast, I3D, C2D, X3D) and Transformers (Uniformer, SwinVideo, TimesFormer, MViT). We then establish two pipelines: training-from-scratch and fine-tuning. We conduct a quantitative evaluation of VCogM and other controlled models on the publicly available HMDB51 dataset. It should be noted that in the fine-tuning pipeline, all models are initialized with weights trained for 300 epochs on the ImageNet1K dataset.

**Part Five**. To validate the generalizability and importance of the proposed PBM, we establish two pipelines: training-from-scratch and transfer learning.

(1) Training-from-scratch pipeline. We follow the embedding method illustrated in Figure [4b](#Fig4), integrating PBM into the VDNN operators from **Part One**. All models are trained on the CIFAR-10 dataset according to the experimental setup outlined in **Part One**. Additionally, PBM is embedded into Swin Transformer, ConvNext, and SMT (representing typical Transformer, CNN, and hybrid CNN-Transformer architectures), training on ImageNet1K, with experimental details following Table [S9](#TS9).

Below is the guideline for integrating PBM into VDNNs. VCNU consists of PBM and P. In VCNU, P is modeled as a combination of convolutional operators with different kernel sizes. Due to the modular design philosophy of CMF, any node in the VCNU computation graph can be replaced with other agency programs. Based on this principle, we simply replace P in VCNU with other agency programs. For example, when integrating PBM into Swin Transformer, use Shifted Window Attention (SWA) as P and connect it with PBM. The output of PBM replaces the output of SWA, while the rest of the structure remains unchanged (Figure [S2a](#FS2)-[c](#FS2)).

Since the introduction of PBM increases the parameter size of Swin, ConvNext, and SMT, we adjust the channel numbers of each model to control the parameter scale, ensuring that any PBM-based model has a similar number of parameters to the original models. This allows for a fair assessment of PBM's actual contribution.

Finally, we use three PBM-based models (Swin-Memory, ConvNext-Memory, SMT-Memory) and their corresponding original models as backbones integrated into the Mask R-CNN object detection framework and UperNet semantic segmentation framework to train on COCO2017 and ADE20K datasets, respectively, to evaluate the advantages of PBM in object detection and semantic segmentation tasks (training details follow Table [S9](#TS9)).

(2) Transfer learning pipeline. In this pipeline, we perform full fine-tuning (FT) and parameter-efficient fine-tuning (PEFT) of Swin-Memory, ConvNext-Memory, SMT-Memory, and the three original control models on both ImageNet1K and CIFAR-100 datasets.

For PEFT, we use Adapter to embed Swin, ConvNext, and SMT as control models. The key difference between FT and PEFT is that FT optimizes all parameters, while PEFT only optimizes the parameters of PBM and Adapter. The experimental setup follows Table [S10](#TS10).

**Table S1**. Statistical results of the UMA on different datasets

| Dataset | Resolution | Function | *n*_1_ | *n*_2_ |
| --- | --- | --- | --- | --- |
| ImageNet1k | 224^2^ | Mean | 20 | 6 |
|  |  | Median | 16 | 4 |
|  |  | Truncated mean | 23 | 7 |
|  |  | Truncated median | 18 | 6 |
| Agri170K | 224^2^ | Mean | 21 | 5 |
|  |  | Median | 18 | 2 |
|  |  | Truncated mean | 27 | 7 |
|  |  | Truncated median | 20 | 6 |
| CIFAR-10 | 224^2^ | Mean | 17 | 6 |
|  |  | Median | 12 | 5 |
|  |  | Truncated mean | 19 | 7 |
|  |  | Truncated median | 13 | 6 |
| CIFAR-100 | 224^2^ | Mean | 14 | 6 |
|  |  | Median | 10 | 4 |
|  |  | Truncated mean | 17 | 7 |
|  |  | Truncated median | 12 | 6 |
| COCO2017 | 1333800 | Mean | 101 | 21 |
|  |  | Median | 60 | 17 |
|  |  | Truncated mean | 88 | 20 |
|  |  | Truncated median | 62 | 18 |
| ADE20K | 512 | Mean | 52 | 12 |
|  |  | Median | 34 | 9 |
|  |  | Truncated mean | 55 | 13 |
|  |  | Truncated median | 38 | 10 |

**Table S2**. Frequency statistics of candidate scenarios in the agricultural sector.

| Rank | Scenario | Utilization Frequency |
| --- | --- | --- |
| 1 | Fruits | 50 |
| 2 | Pests and disasters | 48 |
| 3 | Animals | 42 |
| 4 | Weeds | 29 |
| 5 | Crops | 18 |
| 6 | Vegetables | 16 |
| 7 | Insects | 11 |
| 8 | Crop seeds | 10 |
| 9 | Agricultural machinery | 9 |
| 10 | Agricultural remote sensing | 5 |
| 11 | Soil | 4 |
| 12 | Smart greenhouse | 1 |
| 13 | Agricultural aviation | 1 |

**Table S3**. Category number and actual labeling of Agri170K.

| Scenario | Category | Number | Quantity | Scenario | Category | Number | Quantity |
| --- | --- | --- | --- | --- | --- | --- | --- |
| Animal surveillance | Dog | 1 | 2000 | Vegetable monitoring | Tomato | 57 | 2000 |
|  | Cat | 2 | 2000 |  | Carrot | 58 | 2000 |
|  | Horse | 3 | 2000 |  | Cucumber | 59 | 2000 |
|  | Elephant | 4 | 2000 |  | Pepper | 60 | 2000 |
|  | Cow | 5 | 2000 |  | Potato | 61 | 2000 |
|  | Sheep | 6 | 2000 |  | Eggplant | 62 | 2000 |
|  | Monkey | 7 | 2000 |  | Bell Pepper | 63 | 2000 |
|  | Zebra | 8 | 2000 |  | Onion | 64 | 2000 |
|  | Chicken | 9 | 2000 |  | Cauliflower | 65 | 1713 |
|  | Spider | 10 | 2000 |  | Broccoli | 66 | 1488 |
|  | Lion | 11 | 2000 |  | Yardlong Bean | 67 | 1405 |
|  | Tiger | 12 | 2000 |  | Cabbage | 68 | 1307 |
|  | Panda | 13 | 2000 | Crop monitoring | Ginger | 69 | 2000 |
|  | Hippopotamus | 14 | 2000 |  | Sweet Potato | 70 | 2000 |
|  | Giraffe | 15 | 2000 |  | Corn | 71 | 1879 |
|  | Bear | 16 | 2000 |  | Garlic | 72 | 1613 |
|  | Pig | 17 | 2000 |  | Pea | 73 | 1030 |
|  | Snake | 18 | 2000 |  | others | 74 | 1601 |
|  | Bird | 19 | 2000 | Crop seed testing | Corn | 75 | 1682 |
|  | Leopard | 20 | 1964 |  | Rice(Sprouted) | 76 | 1288 |
|  | Rabbit | 21 | 1924 |  | Soybean | 77 | 920 |
|  | Squirrel | 22 | 1816 |  | Wheat | 78 | 543 |
| Weed management | Deep | 23 | 2000 |  | Rice | 79 | 445 |
|  | Cotton | 24 | 2000 | Pest and disease detection | Tomato | 80 | 2000 |
|  | Marigold | 25 | 2000 |  | Rice | 81 | 2000 |
|  | Corn | 26 | 1735 |  | Apple Leaf | 82 | 2000 |
| Fruit identification | Papaya | 40 | 2000 |  | Grape | 83 | 2000 |
|  | Mango | 41 | 2000 |  | Corn | 84 | 2000 |
|  | Orange | 42 | 2000 |  | Cotton | 85 | 2000 |
|  | Strawberry | 43 | 2000 |  | Various Plants | 86 | 2000 |
|  | Pineapple | 44 | 2000 |  | Strawberry | 87 | 2000 |
|  | Kiwi | 45 | 2000 |  | Potato | 88 | 2000 |
|  | Apple | 46 | 2000 |  | Pumpkin | 89 | 1600 |
|  | Banana | 47 | 2000 |  | Soybean | 90 | 1343 |
|  | Pear | 48 | 2000 |  | Wheat Kernel | 91 | 1317 |
|  | Cantaloupe | 49 | 2000 | Other | Fish | 92 | 948 |
|  | Pomegranate | 50 | 2000 |  | Apple Tree | 93 | 536 |
|  | Grape | 51 | 2000 |  | Flower | 94 | 2000 |
|  | Watermelon | 52 | 2000 |  | Agricultural Machinery | 95 | 2000 |
|  | Pitaya | 53 | 2000 |  | Corn seeding | 96 | 2000 |
|  | Starfruit | 54 | 2000 |  |  |  |  |
|  | Longan | 55 | 2000 |  |  |  |  |
|  | Avocado | 56 | 2000 |  |  |  |  |

**Table S4**. The result of the ablation experiment.

| Model | P | *LTM*-downsampling | UMA-*n* | UMA- *L* | Params  (M) | FLOPs  (M) | Top-1 Acc  (%) |
| --- | --- | --- | --- | --- | --- | --- | --- |
| Step(a) | 1 | Avg | Mean | Linear | 12.5 | 2.2 | 74.52 |
|  | 2 | Avg | Mean | Linear | 12.6 | 2.2 | 74.91 |
|  | 3 | Avg | Mean | Linear | 12.7 | 2.2 | 75.31 |
|  | 4 | Avg | Mean | Linear | 12.7 | 2.3 | 77.54 |
|  | 5 | Avg | Mean | Linear | 12.9 | 2.3 | 77.39 |
| Step(b) | 4 | Avg | Mean | Linear | 12.7 | 2.3 | 77.84 |
| Step(c) | 4 | Avg | Mean | Linear | 12.7 | 2.3 | 77.84 |
|  | 4 | Max | Mean | Linear | 12.7 | 2.3 | 69.7 |
|  | 4 | Conv | Mean | Linear | 12.7 | 2.3 | 77.38 |
| Step(d) | 4 | Avg | Media | Linear | 12.7 | 2.2 | 77.64 |
|  | 4 | Avg | Truncated mean | Linear | 12.8 | 2.3 | 78.14 |
|  | 4 | Avg | Truncated median | Linear | 12.7 | 2.3 | 78.04 |
| Step(e) | 4 | Avg | Truncated mean | Linear | 12.8 | 2.3 | 78.14 |
| Step(f) | 4 | Avg | Truncated mean | Fabnacci | 12.6 | 2.2 | 77.42 |
|  | 4 | Avg | Truncated mean | Piecewise  function | 12.6 | 2.2 | 77.68 |
| Step(g) | 4 | Avg | Truncated mean | Linear | 12.8 | 2.3 | 78.14 |

**Table S5**. The detailed result of Step(b) of the ablation experiment.

| A-aggregation | Params (M) | FLOPs (M) | Top-1 Acc (%) |
| --- | --- | --- | --- |
| Add | 6.2 | 1.2 | 74.15 |
| Hadamard | 6.2 | 1.2 | 0.1 |
| Cat | 12.7 | 2.3 | 77.84 |

**Table S6**. The detailed result of Step(e) of the ablation experiment.

| *n*_1_ | *n*_2_ | Params(M) | FLOPs(M) | Top-1Acc(%) | Top-5 Acc(%) |
| --- | --- | --- | --- | --- | --- |
| √ |  | 12.7 | 2.2 | 77.83 | 93.982 |
|  | √ | 12.5 | 2.2 | 75.25 | 92.709 |
| √ | √ | 12.8 | 2.3 | 78.14 | 94.122 |

**Table S7**. The detailed result of Step(g) of the ablation experiment.

| Label | Memory generation method | Params(M) | FLOPs(M) | Top-1Acc(%) | Top-5 Acc(%) |
| --- | --- | --- | --- | --- | --- |
| Priori | UMA | 12.8 | 2.3 | 78.14 | 94.12 |
| Learnable | Embedding | 12.8 | 2.3 | 77.76 | 93.88 |
| Learnable | Random initialization | 12.8 | 2.3 | 77.92 | 94.04 |

**Table S8**. The config of different scales of VCNU and VCogM.

| Name | Channels | Layers | Layer scale |
| --- | --- | --- | --- |
| VCNU-2.3M | [64, 128, 256, 512] | [1, 1, 1, 1] | 1 |
| VCNU-12M | [96, 192, 384, 768] | [3, 3, 9, 3] | 1 |
| VCNU-21M | [96, 192, 384, 768] | [3, 3, 27, 3] | 1e-4 |
| VCNU-37M | [128, 256, 512, 1024] | [3, 3, 27, 3] | 1e-4 |
| VCogM-25M | [72, 144, 288, 576] | [3, 3, 12, 3] | 1 |
| VCogM-48M | [72, 144, 288, 576] | [4, 4, 32, 4] | 1e-6 |
| VCogM-92M | [96, 192, 384, 768] | [4, 6, 36, 4] | 1e-6 |

**Table S9**. The training setup involves models that follow train-from-scratch on various datasets.

| Config | ImageNet1K/  Agri170K | CIFAR-10 | COCO2017 | ADE20K | HMDB51 |
| --- | --- | --- | --- | --- | --- |
| Method | - | - | Mask R-CNN | UperNet | - |
| Optimizer | AdamW | Adam | AdamW | AdamW | AdamW |
| LR | 1e-3 | 1e-3 | 1e-4 | 6e-5 | 1e-4 |
| Weight decay | 0.05 | - | 0.05 | 0.01 | 0.05 |
| Beta | (0.9, 0.999) | (0.9,0.999) | (0.9, 0.999) | (0.9, 0.999) | (0.9, 0.999) |
| Batch size | 1024 | 1024 | 16 | 16 | 32 |
| Frame number/ Sampling rate | - | - | - | - | 16/4 |
| LR schedule | cosine | linear | steps: [27, 33] (3×) | linear:  160k | cosine |
| Minimum learning rate | 1e-5 | - | - | 0.0 | 1e-6 |
| Warmup epochs | 20 | - | - | 1.5k | 10 |
| Warmup learning rate | 1e-6 | - | - | 1e-6 | 1e-6 |
| Training epochs | 300 (ImageNet1K)/  120 (Agri170K) | 120 | 36 (3×) | 160k | 200 |
| Augmentation | Rand-m9-mstd0.5-inc1 | - | - | - | Rand-m5-mstd0.25 |
| Color jitter | 0.4 | - | - | - | 0.4 |
| Mixup α | 0.8 | - | - | - | 0.8 |
| Cutmix α | 1.0 | - | - | - | 1.0 |
| Random erasing | 0.25 | - | - | - | - |
| Label smoothing | 0.1 | - | - | - | 0.1 |
| Gradient clip | 5.0 | - | - | - | 20.0 |
| Drop path | [0.2, 0.3, 0.5] (VCogM)  [0.1, 0.2, 0.3] (VCNU) | - | [0.2, 0.3, 0.5] (VCogM)  [0.1, 0.2, 0.3] (VCNU) | [0.2, 0.3, 0.5] (VCogM)  [0.1, 0.2, 0.3] (VCNU) | 0.1 |
| Layer scale value | [1, 1e-6, 1e-6] (VCogM)  [1, 1e-4, 1e-4] (VCNU) | - | [1, 1e-6, 1e-6] (VCogM)  [1, 1e-4, 1e-4] (VCNU) | [1, 1e-6, 1e-6] (VCogM)  [1, 1e-4, 1e-4] (VCNU) | 1 |

**Table S10**. The training setup encompasses models that follow transfer learning on different datasets.

| Config | ImageNet1K | CIFAR-100 | HMDB51 |
| --- | --- | --- | --- |
| Method | - | - | - |
| Optimizer | AdamW | SGD | AdamW |
| LR | 2e-5 | 0.01 | 5e-5 |
| Weight decay | 0.05 | - | 0.05 |
| Beta | (0.9, 0.999) | - | (0.9, 0.999) |
| Momentum factor | - | 0.9 | - |
| Batch size | 512 | 512 | 32 |
|  |  |  | 16/4 |
| LR schedule | cosine | cosine | cosine |
| Minimum learning rate | 2e-7 | - | 1e-6 |
| Warmup epochs | 5 | 5 | 5 |
| Warmup learning rate | 2e-8 | 1.0 | 1e-6 |
| Training epochs | 30 | 10 | 30 |
| Augmentation | Rand-m9-mstd0.5-inc1 | - | Rand-m5-mstd0.25 |
| Color jitter | 0.4 | - | 0.4 |
| Mixup α | 0.8 | - | 0.8 |
| Cutmix α | 1.0 | - | 1.0 |
| Random erasing | 0.25 | - | - |
| Label smoothing | 0.1 | - | 0.1 |
| Gradient clip | 5.0 | - | 20.0 |
| Drop path | [0.2, 0.3, 0.5] (VCogM)  [0.1, 0.2, 0.3] (VCNU) | - | 0.1 |
| Layer scale value | [1, 1e-6, 1e-6] (VCogM)  [1, 1e-4, 1e-4] (VCNU) | - | 1 |


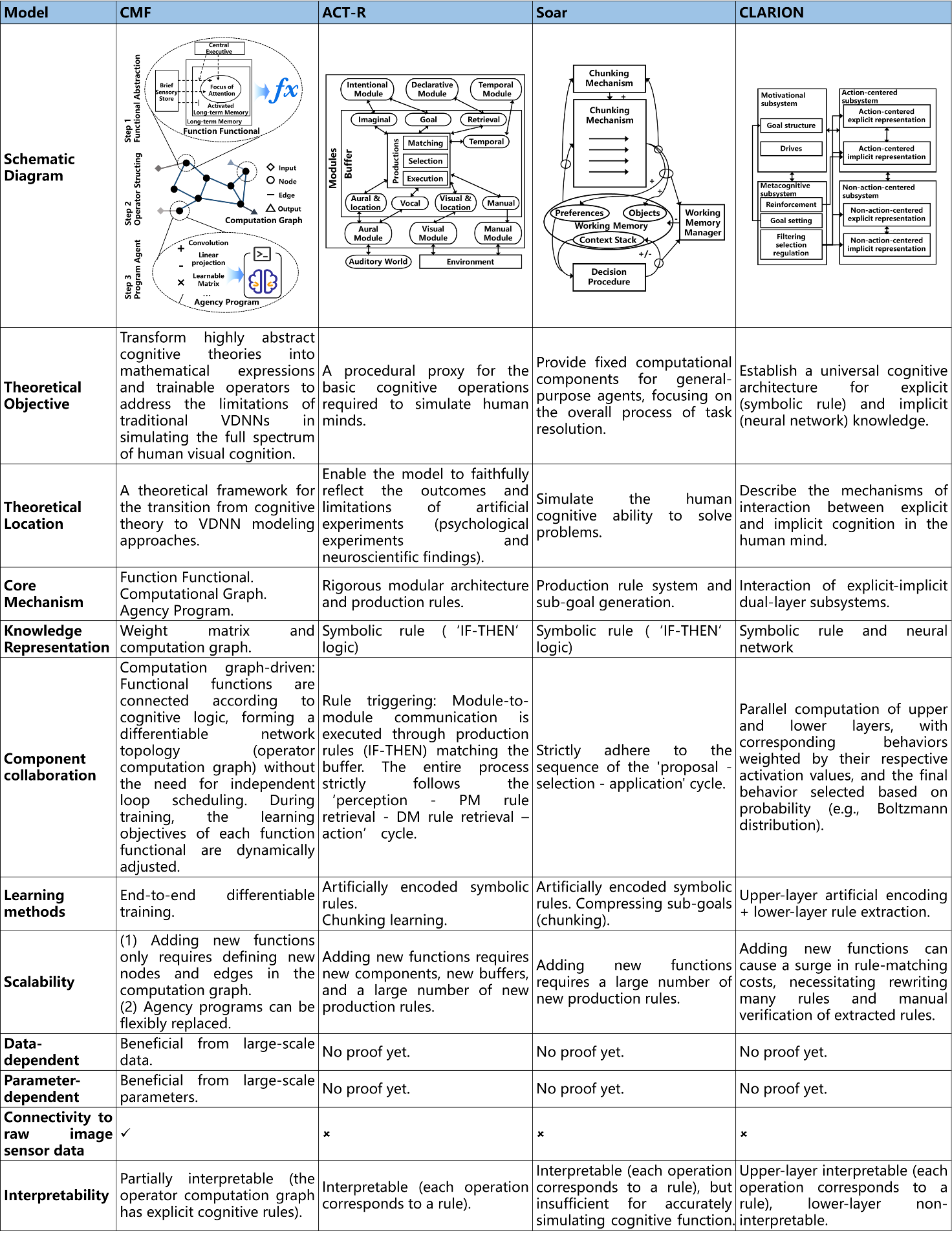


**Figure S1.** Additional illustration of an explicit comparison of CMF with classical cognitive architectures (models).


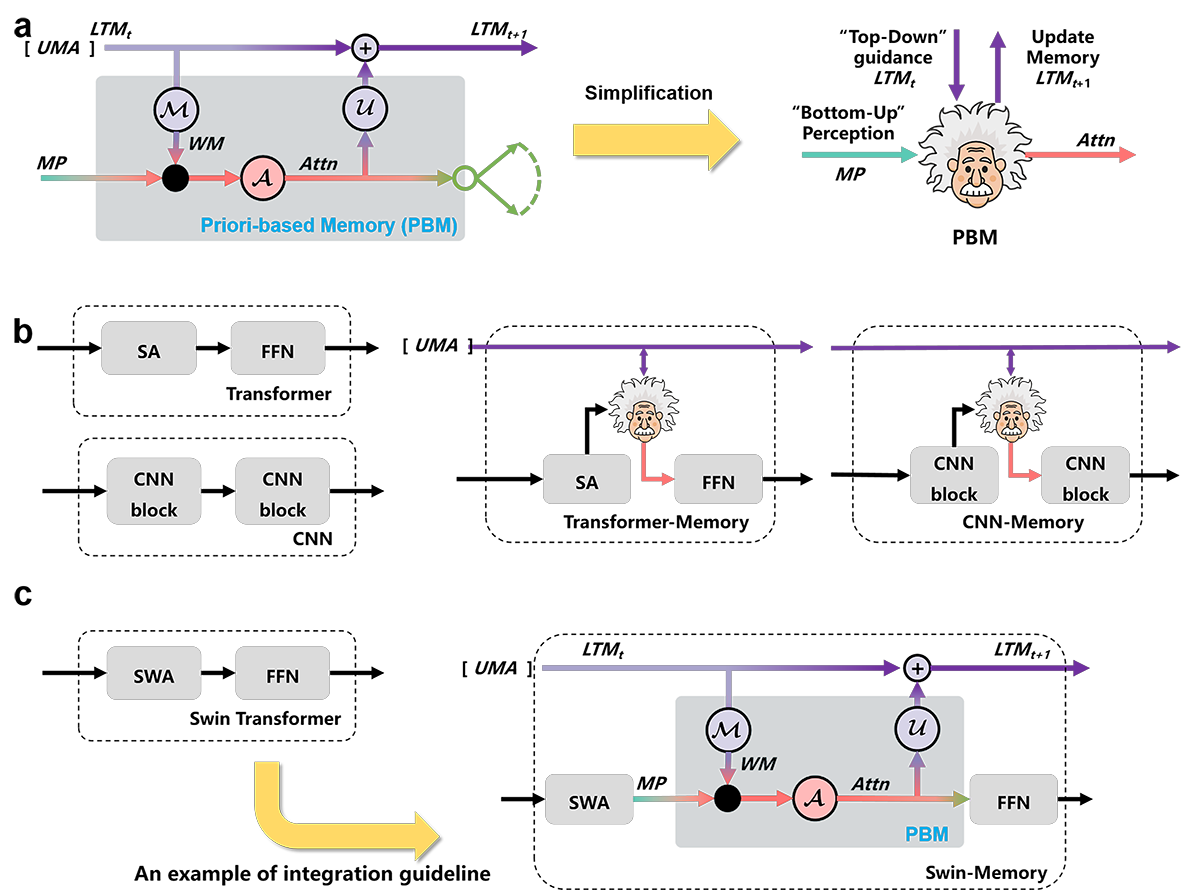


**Figure S2.** The integration guidelines of PBM for mainstream architectures. a) Simplified illustration of PBM. b) An example of PBM embedded within Transformer and CNN architectures. c) An example of integration guideline of PBM embedded within Swin Transformer.

**Table S11.** Experimental results of introducing PBM within mainstream architectures on the recognition accuracy and inference speed. All inference speeds were measured by calculating 100 images per run on a single RTX 4090 or Intel 8352V, with the results averaged over 10 runs.

| Model | Using Memory | Istropic | Parameters (M) | FLOPs (G) | Top-1 Accuracy (%) | GPU Inference Times  (Mean±STD) (Images/s) | CPU Inference Times  (Mean±STD) (Images/s) |
| --- | --- | --- | --- | --- | --- | --- | --- |
| Swin | 🗶 | 🗶 | 28.3 | 4.5 | 81.18 | 108.844±0.020 | 29.107±1.640 |
|  | ✓ | 🗶 | 32.8 | 5.2 | 81.46 | 42.020±0.021 | 4.487±0.083 |
|  | ✓ | ✓ | 28.9 | 4.5 | 81.42 | 42.374±0.018 | 4.629±0.142 |
| SMT | 🗶 | 🗶 | 20.5 | 4.7 | 83.53 | 37.043±0.013 | 10.207±0.067 |
|  | ✓ | 🗶 | 26.5 | 5.5 | 83.77 | 22.03±0.001 | 3.566±0.056 |
|  | ✓ | ✓ | 20.5 | 4.3 | 83.68 | 22.024±0.007 | 3.506±0.034 |
| ConvNext | 🗶 | 🗶 | 29 | 4.5 | 82.1 | 196.267±0.262 | 25.531±0.096 |
|  | ✓ | 🗶 | 35.5 | 5.6 | 82.31 | 45.019±0.020 | 4.124±0.014 |
|  | ✓ | ✓ | 28.6 | 4.6 | 81.75 | 45.851±0.026 | 4.484±0.111 |

**Table S12.** Experimental results of introducing PBM and Adapter within mainstream architectures on the recognition accuracy and inference speed. All inference speeds were measured by calculating 100 images per run on a single RTX 4090 or Intel 8352V, with the results averaged over 10 runs.

| Model | Transfer Learning Method | Parameters (M) | FLOPs (G) | ImageNet1k  Top-1 Accuracy (%) | CIFAR-100  Top-1 Accuracy (%) | GPU Inference Times  (Mean±STD) (Images/s) | CPU Inference Times  (Mean±STD) (Images/s) |
| --- | --- | --- | --- | --- | --- | --- | --- |
| Swin | Adapter | 91.2 | 15.8 | 84.06 | 77.02 | 48.637±0.063 | 12.540±0.030 |
|  | Memory | 102.4 | 17.8 | 84.14 | 77.93 | 28.275±0.005 | 3.587±0.030 |
|  | No UMA | 102.4 | 17.8 | - | - | 40.662±0.422 | 10.311±0.224 |
| SMT | Adapter | 84 | 18.2 | 86.03 | 22.32 | 21.771±0.015 | 5.009±0.011 |
|  | Memory | 95.1 | 20.3 | 86.23 | 79.33 | 16.485±0.004 | 2.563±0.011 |
|  | No UMA | 95.1 | 20.3 | - | - | 19.239±0.043 | 4.565±0.005 |
| ConvNext | Adapter | 93.8 | 16.3 | 85.33 | 77.2 | 71.764±0.122 | 12.224±0.057 |
|  | Memory | 110.6 | 19.4 | 85.4 | 77.31 | 32.895±0.012 | 3.778±0.039 |
|  | No UMA | 110.6 | 19.4 | - | - | 49.246±1.525 | 9.239±0.030 |
